# Scoping review on vector-borne diseases in urban areas: transmission dynamics, vectorial capacity and co-infection

**DOI:** 10.1186/s40249-018-0475-7

**Published:** 2018-09-03

**Authors:** Marcus Eder, Fanny Cortes, Noêmia Teixeira de Siqueira Filha, Giovanny Vinícius Araújo de França, Stéphanie Degroote, Cynthia Braga, Valéry Ridde, Celina Maria Turchi Martelli

**Affiliations:** 1 Public Health England Sierra Leone Country Office, Freetown, Sierra Leone; 2 Aggeu Magalhaes Institute (IAM) / Oswaldo Cruz Foundation (Fiocruz), Avenida Professor Moraes Rego, s/n. Cidade Universitaria. CEP 50, Recife, Pernambuco 740-465 Brazil; 3 Universidade de Pernambuco (UPE), Recife, Pernambuco Brazil; 4 Liverpool School Of Tropical Medicine (LSTM), London, UK; 5 Secretariat of Health Surveillance, Ministry of Health, Brasilia, Brazil; 6 University of Montreal School of Public Health (ESPUM), Montreal, Quebec Canada; 7 IRD (French Institute For Research on Sustainable Development), CEPED (IRD-Université Paris Descartes), Universités Paris Sorbonne Cités, ERL INSERM SAGESUD, Paris, France

**Keywords:** Arboviruses, Disease vectors, Coinfection, Urban population, Epidemiology, Review

## Abstract

**Background:**

Transmission dynamics, vectorial capacity, and co-infections have substantial impacts on vector-borne diseases (VBDs) affecting urban and suburban populations. Reviewing key factors can provide insight into priority research areas and offer suggestions for potential interventions.

**Main body:**

Through a scoping review, we identify knowledge gaps on transmission dynamics, vectorial capacity, and co-infections regarding VBDs in urban areas. Peer-reviewed and grey literature published between 2000 and 2016 was searched. We screened abstracts and full texts to select studies. Using an extraction grid, we retrieved general data, results, lessons learned and recommendations, future research avenues, and practice implications. We classified studies by VBD and country/continent and identified relevant knowledge gaps. Of 773 articles selected for full-text screening, 50 were included in the review: 23 based on research in the Americas, 15 in Asia, 10 in Africa, and one each in Europe and Australia. The largest body of evidence concerning VBD epidemiology in urban areas concerned dengue and malaria. Other arboviruses covered included chikungunya and West Nile virus, other parasitic diseases such as leishmaniasis and trypanosomiasis, and bacterial rickettsiosis and plague. Most articles retrieved in our review combined transmission dynamics and vectorial capacity; only two combined transmission dynamics and co-infection. The review identified significant knowledge gaps on the role of asymptomatic individuals, the effects of co-infection and other host factors, and the impacts of climatic, environmental, and socioeconomic factors on VBD transmission in urban areas. Limitations included the trade-off from narrowing the search strategy (missing out on classical modelling studies), a lack of studies on co-infections, most studies being only descriptive, and few offering concrete public health recommendations. More research is needed on transmission risk in homes and workplaces, given increasingly dynamic and mobile populations. The lack of studies on co-infection hampers monitoring of infections transmitted by the same vector.

**Conclusions:**

Strengthening VBD surveillance and control, particularly in asymptomatic cases and mobile populations, as well as using early warning tools to predict increasing transmission, were key strategies identified for public health policy and practice.

**Electronic supplementary material:**

The online version of this article (10.1186/s40249-018-0475-7) contains supplementary material, which is available to authorized users.

## Multilingual abstracts

Please see Additional file 1 for translations of the abstract into the six official working languages of the United Nations.

## Background

According to the World Health Organization (WHO), vector-borne diseases (VBDs) account for more than 17% of all infectious diseases and cause more than 1 million deaths annually [1]. Vector-borne diseases are transmitted from person to person via a competent vector, such as mosquitoes, midges and flies.

Transmission dynamics describes a range of factors influencing how effectively transmission occurs over space and time, and in a specific population. These factors include basic reproduction number, host immunity, travel and human behaviour. Transmission dynamics are determined by the interaction between pathogen, vector, host (human, and in many cases also other animals, serving as reservoir or amplifier) and other environmental factors [2].

Vectorial capacity refers to the ability of a mosquito population’s to transmit the pathogen to a new susceptible population [3].

The term co-infection describes human infection through more than one organism, either by different strains of the same (e.g. two genetically different falciparum malaria protozoa), or entirely different pathogens (e.g. falciparum malaria protozoa and intestinal helminths). Here, also co-circulation is considered, when more than one different pathogens are present in an insect vector (e.g. in mosquito populations of a specific region) [4].

Malaria is a VBD that caused over 400 000 deaths in 2015, most of them in children under 5 years of age [5]. Traditionally associated with rural transmission, malaria is increasing found in urban and peri-urban areas [6, 7]. An entomological marker of malaria transmission is the entomological inoculation rate (EIR). It describes the number of infected bites per unit of time, and a function of the so-called ‘man biting rate’ (MBR, the number of bites per person per time unit) and the sporozoite rate (rate of infected mosquitoes, i.e. those carrying malaria parasites ready to infect humans).

Currently dengue, a virus transmitted through *Aedes* mosquitoes, threatens a half-billion people globally [8]. Unlike yellow fever, where sylvatic (forest) mosquito species and non-human primate reservoirs play a critical role in the transmission, dengue only requires humans, a fact that explains its rapid spread in populated urban areas [9]. Dengue incidence has increased dramatically in the Americas, and recent introductions of chikungunya and Zika have resulted in serious epidemics in these regions [10, 11]. Other VBDs, such as American trypanosomiasis (Chagas disease), leishmaniasis, and filariasis, have affected hundreds of millions of people globally [12].

Approximately half of the world’s population currently live in cities. The United Nations projects that 2.5 billion people will be added to the urban population by 2050, mostly on the Asian and African continents [13]. This rapid and increasing urbanization has posed a great challenge to nations, especially those less developed [14]. Urbanization has had an impact on the epidemiological pattern of infectious diseases. The main factors are urban sprawl into forested areas, overcrowding, and precarious urban infrastructures and housing in urban areas of developing countries. The absence of necessary investments in infrastructure in these countries poses a serious threat to human health, including the (re-)emergence and adaptation of infectious agents in urban areas such as dengue in South East Asia or, Chagas in Latin America in areas where poor housing is hindering effective vector control [14–16].

Basic knowledge about VBD transmission includes population susceptibility, vectorial capacity, and interaction of infectious agents. The understanding of VBD transmission and persistence is essential for establishing effective prevention and control interventions. Of similar importance is to know key aspects of introduction, maintenance, and spread of VBDs, as well as the role of environmental and climate factors, the urbanization process, socioeconomic conditions, population dynamics and mobility [2, 17–20].

This scoping review evaluated the current state of knowledge on transmission dynamics, vectorial capacity, and co-infection regarding VBDs in urban areas from 2000 to 2016, to identify research gaps and implications for public health policy and practice.

## Main text

### Research question

We conducted a scoping review adapting Arksey and O’Malley’s [21] methodological framework. A three-round eDelphi survey was used to select six topics considered highest priority by a panel of 109 international VBD experts, the majority of them being from Brazil, Burkina Faso, Canada, Colombia, France, Spain and the United States of America (43% researchers; 52% public health decision-makers; 5% from the private sector). The three rounds were: 1) suggestions of research topics; 2) ranking of topics identified (more than 80 topics, rated from “1–eliminate” to “5–top priority”); and 3) final selection of highest-priority topics (the 20 subjects rated 4 or 5 by more than 65% of the participants). By the end of the third round, the present topic—the impact of transmission dynamics, vectorial capacity, and co-infections on the burden of vector-borne diseases in urban areas—had obtained the mean rating of 3.90 ± 0.92 and was ranked fourth. It was therefore among six top rated topics taken forward for research carried out by the consortium groups.

### Search strategy

We used the following key concepts: [“transmission dynamics” OR “vectorial capacity” OR “co-infection”] AND “vector-borne” AND “urban areas” AND “epidemiology”. All possible word variations and MeSH terms (as appropriate) were added to the search command and validated by a librarian (see Additional file 2) for the following databases: PubMed, Embase, Global Health, Cochrane Database of Systematic Reviews, OpenGrey, the Grey Literature Report, and WHOLIS. Additional articles were identified by screening the references of papers that met our inclusion criteria. As part of the protocol development the consortium members considered the 2014 World Urbanization Prospects issued by the Population Division of UNDESA [13].

The literature search was undertaken from August to September 2016. We used Mendeley and Endnote software to manage references and remove duplicates.

### Inclusion and exclusion criteria

We included all articles and reports published in peer-reviewed journals or grey literature written in English, French, Portuguese, Spanish, German, or Italian and published between 2000 and 2016. We excluded: articles focused on clinical or laboratory characteristics, vector prevalence or seroprevalence only; reviews; conference papers; articles without research data; articles not addressing human disease; articles reporting water-borne diseases or diseases without insect vector; studies conducted in rural areas; and interventional studies, such as mass drug administration, intermittent preventive treatment, and vector control programs.

### Study selection

We performed a pilot round of study selection to evaluate consistency in the application of the above criteria and discuss discrepancies with 20 randomly selected references. For both abstract and full-text screening, two independent reviewers (FC and NTSF) selected the studies through the title and abstract/full-text, and a third reviewer (ME) resolved discordances.

After completing full-text screening for 205 articles, an additional step was introduced to retain references that combined at least two elements of the search strategy: transmission dynamics and vectorial capacity or transmission dynamics and co-infection. This last step was done manually by the reviewers.

### Data extraction, summary, and analysis

An extraction grid was created allowing to record *for each* of the selected studies the following information: general information, key objectives and methods; overview of results; methodological limitations and challenges encountered in lessons learnt/recommendations; future research avenues; and, public health policy or practice implications. Similarly, the methodological and quality aspects of each study were evaluated using the modified Mixed Methods Appraisal Tool (MMAT; for description of qualitative, quantitative, and mixed methods studies) [22] and parts of the TIDieR (Template for Intervention Description and Replication) checklist [23]. Summary tables and graphs were produced. Initially, the three contributors (FC, ME, NTSF) independently extracted data from the same five articles, to ensure harmonization. Any remaining difficulties were resolved in a discussion with the remaining two participants. Subsequently, the remaining 45 articles were summarised with quality assessed by the same three contributors and results recorded in the extraction grid.

## Results

### Description of included studies and their funding sources

**Fig. 1:**
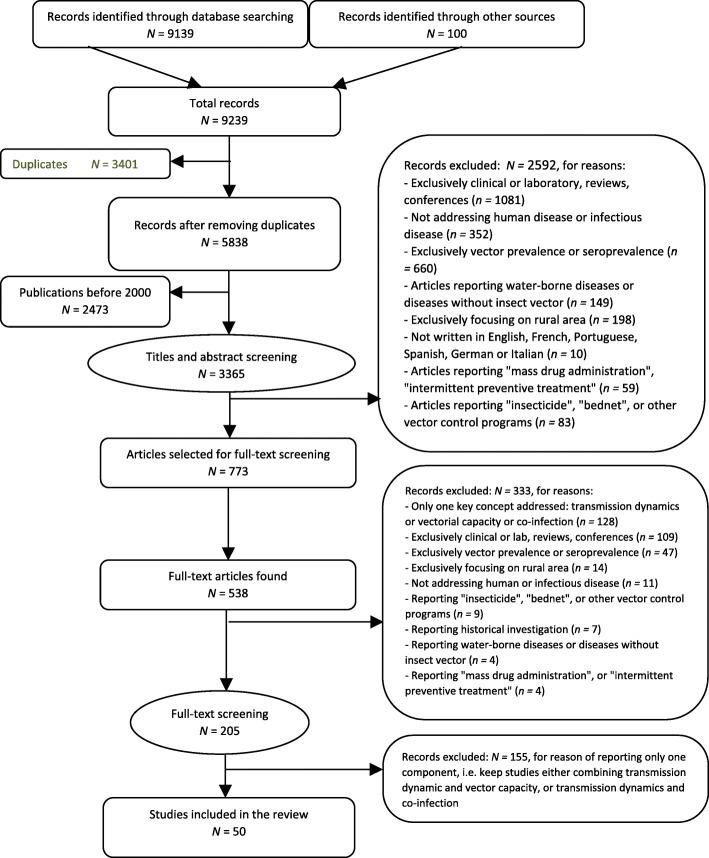
Prisma Chart showing references retrieved at different stages of the search (full text review)

The search strategy initially identified 9239 records. After removing duplicates and articles published before 2000, we screened 3365 articles by title and abstract and retrieved 773 of them. After full-text screening, 50 articles were selected for the scoping review (Preferred Reporting Items for Systematic Reviews and Meta-Analyses [PRISMA] flowchart, Fig. 1).

**Fig. 2:**
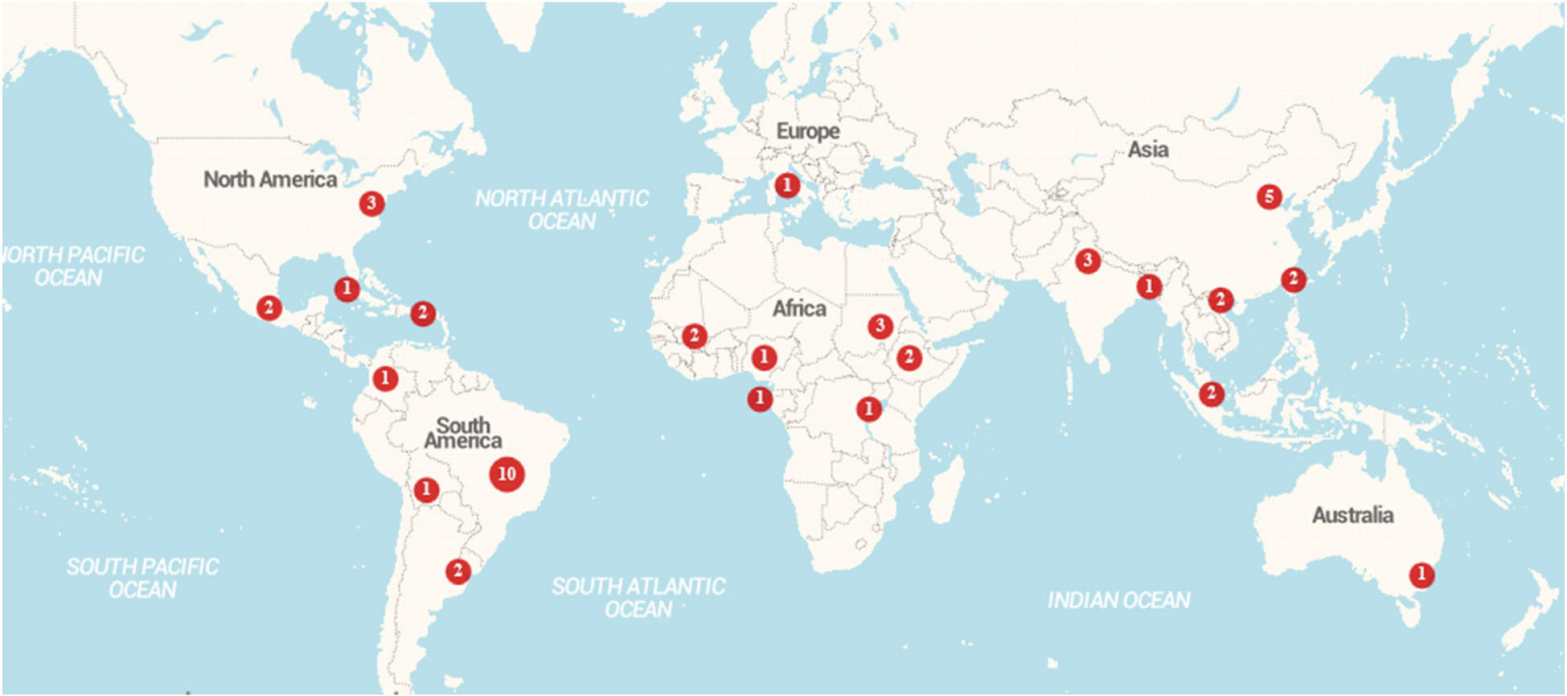
Distribution of final selection of *N =* 50 references by geographic regions

Most of the 50 studies retained were conducted in the Americas (*n =* 23; 46%), followed by Asia (*n =* 15; 30%), Africa (*n =* 10; 20%), Europe (*n =* 1; 2%) and Australia (*n =* 1; 2%) (continents, Table 1; countries, Additional file 3; map, Fig. 2). Selected articles were organized into three groups of diseases: 1) dengue (*n =* 20; 40%), 2) malaria (*n =* 15; 30%), and 3) others (*n =* 15; 30%), which included parasitic diseases: leishmaniasis (*n =* 4) and Chagas disease (*n =* 2); other arboviruses: chikungunya (*n =* 2), West Nile virus (*n =* 2), yellow fever (*n =* 2), and Ross River virus (*n =* 1); and two bacterial diseases: plague (*n =* 1) and rickettsiosis (*n =* 1) (Table 1). Two studies reported on co-infections, one on multiple *Plamodium falciparum* strains, the other on combined malaria, helminth, and human immunodeficiency virus (HIV) infection in pregnant women. Studies are summarized in Table 2.

**Table 1:** Final selection of N = 50 references: Group of diseases: dengue, malaria and others (ordered by parasitic, viral and bacterial diseases) by continent

Continent	Dengue	Malaria	Leishmaniasis	Chagas disease	West Nile	Chikungunya	Yellow fever	Ross River virus	Plague	Rickettsiae	Total
Americas	12	2	3	2	2	0	1	0	0	1	23
Europe	0	0	0	0	0	1	0	0	0	0	1
Africa	1	8	0	0	0	0	1	0	0	0	10
Asia	7	5	1	0	0	1	0	0	1	0	15
Australia	0	0	0	0	0	0	0	1	0	0	1
Total	20	15	4	2	2	2	2	1	1	1	50

**Table 2:** Description of included studies, by disease

Authors, Date	VBD Country Region City	Study type	Objectives (outcomes measures)	Results and conclusions	Learning points and recommendations: transmission dynamics, vectorial capacity, co-infection	Main limitations, comments	Future research, public health policy & practice	Funding
Dengue								
AMERICAS								
Araujo et al., 2015 [39]	Dengue Brazil Southeast São Paulo	Spatial modelling	To assess incidence of dengue and its association with multiple environmental and socioeconomic factors	Dengue incidence was higher in areas of high land surface temperature (28–32 °C) and in slum-like areas. Most cases occurred in areas with low vegetation cover. Laboratory vector experiments indicated that higher temperature was favourable for *Ae. aegypti* proliferation.	Incidence mostly affected by land surface temperature (LST), due to *Ae. aegypti* proliferation. Recovering vegetation on urban heat island (UHI).	None reported.	Importance of evaluating LST (environmental inner city conditions), slum-like areas and human activities (man-made reservoirs) for DENV transmission. Translational study in dengue.	NR
Dibo et al., 2008 [35]	Dengue Brazil Southeast Mirrasol		*Ae. aegypti* female abundance (collection) and eggs (oviposition traps); sensitivity of both methods; correlation of entomological indices (positivity and averages of females and eggs) dengue incidence and climate.	Almost 20 000 specimens of culicids were caught, ~ 10% were *Ae. aegypti*. The ovitrap caught 165 000 eggs in the period and 173 autochthonous cases of dengue were recorded.	Relationship between climatic factors, vector and disease. Use of information about climate for early detection of epidemics and for establishing more effective prevention strategies.	Using information about reported dengue fever (DF) cases may represent only part of the total number of dengue virus infections, rainfall data were obtained for a location not exactly at the mosquito collection site, and temperatures were available only for a neighbouring city.	Widespread application in planning and undertaking surveillance and dengue control activities	NR
Teixeira et al., 2011 [41]	Dengue Brazil Southeast Rio de Janeiro	Ecologic spatial modelling	Dengue spatial distribution and its relationship to environmental and socioeconomic variables	Direct association was found between dengue incidence and rainfall, in both final generalized linear model and some monthly CAR models. Direct association was also found between dengue incidence and a 1-month rainfall time lag.	Significant direct association between dengue incidence and rainfall +lag, Gini index, and Breteau index for *Ae. albopictus*, but only the Gini index showed strong association. Importance of socio-environmental variables in the dynamics of dengue transmission.	A methodological limitation was data georeferencing, due to the incomplete notification data. Use of Breteau index as an indicator of vector infestation since it was considered precarious and not a reliable indicator.	Need to investigate and analyze the association between dengue incidence and explanatory variables through more complex models (complete Bayesian model) capable of simultaneously capturing the spatial and temporal autocorrelation structures.	NR
Corrêa et al., 2005 [34]	Dengue Brazil Southeast Belo Horizonte	Ecologic October 1997– May 2001	Link between vector infestation index and dengue incidence	*Ae. aegypti* infestation was positively associated with dengue incidence.	Higher building infestation rates were associated with higher risk of disease. This correlation was significant but weak.	Building infestation rates have limitations for estimating vector infestation.	To reduce and maintain the house infestation index below the 1% threshold.	NG
Teixeira et al., 2002 [25]	Dengue Brazil Northeast Salvador	Prospective populational study, seroprevalence and incidence after 1 year of follow-up	Relationship between intensity of viral circulation and standard of living, environmental quality and vector density	Wide range of prevalence of previous dengue infection (16.2% to 97.6%) in the area. Areas with a lower prevalence at baseline presented higher incidence of dengue infection. High seroincidence was observed in areas with low vector indices.	Increase in herd immunity to two different serotypes of dengue, and maintenance of the environmental conditions necessary and sufficient for transmission of new serotypes, created conditions for occurrence of epidemic haemorrhagic dengue.	Some years after the virus’ introduction, transmission continues to occur, but with a decrease in the perception of the problem by individuals and health professionals and hence reduced sensitivity of the surveillance system.	Further studies are required to identify the behavioural and environmental risk factors (public and private domains) that have the greatest influence on transmission.	NG
Méndez et al., 2006 [26]	Dengue Colombia Valle del Cauca Cities: Cali, Palmira, Tulua, Buenaventura	Prospective study	Study dynamics during and after epidemics; mosquito infection rates, cases, serotypes (infection rates)	Infections in students: asymptomatic cases outnumber symptomatic (silent circulation of DENV). Infections in mosquitoes: *Ae. aegypti* adult mosquito and larvae house indexes were not found to be associated with increased burden of disease.	Virological surveillance for detecting infected *Aedes* mosquitoes as early warning system for outbreaks.	Asymptomatic infections potential source of subsequent outbreaks; to include pooled mosquito infection rates in entomologic surveillance.	Need to understand a) inter-outbreak virus circulation and b) *Ae. albopictus* vectorial capacity.	NG
Estallo et al., 2014 [29]	Dengue Argentina Cordoba	Descriptive	Spatio-temporal dynamics of DF outbreak 2009	Spatio-temporal cluster of dengue cases. Outbreak started because of imported cases from neighbouring provinces. *Ae. aegypti* infestation levels were not associated with occurrence of the cases.	Significant risk factor for dengue emergence: travel, migration and displacement within and outside the country. Dengue virus spread may be related to human movements.	Applications of vector control measures (focal intervention at dengue positive houses) may have interrupted DENV transmission.	Need to develop innovative strategies of vector control and arboviral surveillance to prevent future outbreaks.	NG, U
Martinez-Vega et al., 2015 [26]	Dengue Mexico City 2 endemic communities	Prospective cohort with spatial modelling	To determine risk in individuals living near dengue cases (exposed cohort); vector data	At baseline, seroprevalence of infection was ~ 44% (5–14 yrs) and 92% (> 35 years) indicating high exposure levels. 3-fold increased risk of dengue infection among the exposed cohort.	Asymptomatic subjects could participate in transmission; peri-domestic transmission was important for ~ 3 months.	Spread of infection within the community mainly depended on human mobility; limited role of vector (50-m ratio) for dengue spread.	Clarify the role of asymptomatic individuals in transmission. Private health centres should contribute to notifications.	NG, PI
Reiter et al., 2003 [37]	Dengue USA: Laredo Mexico: Nuevo Laredo	Two serosurveys	Investigations of 1999 outbreak affecting cross border urban area of Laredo, USA, and Nuevo Laredo, Mexico.	Incidence of dengue was higher in Mexico (16%) than on USA side (1.3%). Vector was more abundant in Texas where dengue transmission was lower.	Prevalence of dengue in Texas was primarily due to economic, rather than climatic, factors.	Need to explore role of other animals as feeding source for *Aedes* mosquitoes.	Growing economy supporting use of air conditioning likely to decrease dengue rates.	NG
Barrera et al., 2011 [32]	Dengue Puerto Rico San Juan	Longitudinal modelling study	Influence of weather and human factors on *Aedes* vector and cases (numbers)	Containers for water storage and discarded tires were considered important breeding sites. Peak in mosquitoes’ density preceded increase in dengue incidence.	Weather and human factors driving *Ae. aegypti* dynamics and oviposition.	Passive surveillance system captures small number of symptomatic cases and misses most asymptomatic ones.	Consider using ovitraps for surveillance.	IG
Sanchez et al., 2006 [33]	Dengue Cuba Havana	Case control study	Study *Aedes* larval indices and risk of Havana outbreak (2000)	Effect of Breteau index (BI) within a radius of 100 m at 2-month intervals. Larval indices used in study predicted transmission with 78% sensitivity and 63% specificity.	Analysis of BI at local level, with human defined boundaries, could be introduced in control programs to identify high risk areas.	Underestimating larvae; increased capture after dengue reported; ‘missing’ cases reported; lack of transferability to other epidemic settings or locations.	Similar studies in future epidemics and in other settings are necessary to verify general applicability.	NG, IG
Fouque et al., 2004 [40]	Dengue French Guiana	Descriptive	Epidemiology of dengue after the 1st epidemic of dengue hemorrhagic fever (DHF) (1991–1993) and during endemic period (1993–1995)	DENV1, 2 and 4 were isolated from dengue cases. DENV4 from mosquitoes. Widespread circulation of DENV in urban and rural areas. Small outdoor containers were most common vector breeding grounds.	Vertical transmission of DENV indicated that the vector can be a reservoir of dengue virus.	Females deposit small numbers of eggs in several breeding containers. Not possible to estimate fluctuations in *Ae. aegypti* populations.	Understanding of endemic circulation of dengue is a prerequisite for development of a dengue early warning system.	NG
ASIA								
Wu et al., 2007 [43]	Dengue China Taiwan Kaohsuing	Time series modelling	Association between weather variability and dengue fever	Incidence of DF was negatively associated with monthly temperature deviation and relative humidity; time lag of 2 months.	Weather variables (temperature and humidity) were significantly associated with dengue incidence rate. Vector density did not appear to be a good predictor of disease occurrence.	Change in: disease diagnostic or classification criteria, urbanization status/land use, vector control program, and personal protection.	Include development and verification of analytical models appropriate for predicting influences of global warming on changes in disease patterns at regional level.	NG
Chang et al., 2015 [42]	Dengue Chian Taiwan Kaohsuing, Tainan (non-endemic areas)	Time series regression modelling study	Temporal relation between local weather, entomology, and confirmed cases	Risk of increased number of cases was associated with entomologic indices on a lag of 2 weeks or 1 month.	Proposed 2-stage model suitable to identify best set of lag effects to generate outbreak prediction.	Complex relation between human hosts, environmental factors and dynamic changes in mosquito density. Difficult to extrapolate to other settings with distinct weather conditions, human immunity profiles and vector distributions.	Suggestion for other countries to apply the authors’ proposed 2-stage modelling system.	NG
Ali et al., 2003 [36]	Dengue Bangladesh Dhaka	Spatial modelling study	Association of spatial clusters and vector during large outbreak (distribution; relative risk)	Dengue clusters far less observed in major hospitals than within or around the households; importance of *Ae. albopictus* in the transmission.	Case clusters and *Ae. albopictus* were linked in densely populated area.	Hospital-based surveillance underestimates disease burden; self-reporting cases limit precision.	Controlling the *Aedes* mosquito; improving case management.	NG, IG, NGO
Anders et al., 2015 [28]	Dengue Vietnam Ho Chi Minh City	Prospective spatial modelling study	Distribution of dengue risk (prevalence, odds ratios)	At baseline, there was 2-fold higher risk in case clusters than controls. Follow-up (14 days) suggested no excess risk for dengue infection in clusters. Prevalence of DENV infection in *Ae. aegypti* was similar in cases and control houses, and low (1%).	Evidence for some household dengue risk clustering, on short temporal scale rather than sustained chains of localized transmission.	Short follow-up: longer focal transmission chains missed? Self-reporting: underestimating symptomatic infections? Vector control applied after onset of illness causing reduced clustering observed?	Reactive peri-focal insecticide spraying unlikely to prevent many additional infections.	U
Yang et al., 2009 [30]	Dengue China Cixi	Descriptive of dengue outbreak	Factors determining the dengue outbreak and measures to avoid additional epidemic outbreaks	Dengue incidence had no relationship with local meteorological factors. *Ae. albopictus* was the main vector.	Social factors and hygienic conditions for endemic villagers and immigrant workers.	Not reported.	Public education to include handling of environmental factors (artificial containers with stagnant water). Socioeconomic conditions should be taken into account to interrupt transmission.	NG
Peng et al., 2012 [31]	Dengue China Dongguan	Descriptive of outbreak	Describe epidemic, risk factors and control measures	Attack rate: ~ 51/100000 *Ae. albopictus* was only vector species responsible for the outbreak. DENV1 serotype. Dengue outbreak initiated by imported case from Southeast Asia.	Urbanization, population density, habitats and habits favouring *Ae. albopictus*; high infestation, poor housing.	Underreporting and/or misclassification of dengue.	Importance of a surveillance system for infectious diseases control. Surveillance needed in rapidly urbanized areas and among immigrants.	NG
Sang et al., 2014 [24]	Dengue China Guangzhou	Modelling study	Predicting local dengue transmission in Guangzhou, China, influence of imported cases, vector density, climate variability	Seasonal pattern. Imported dengue cases, mosquito density and climate variability are important in dengue transmission.	Imported DF cases, mosquito density were critical for DENV transmission.	Relation between herd immunity, mosquito-human interaction, virus strain and mosquito daily survival rate.	Establishment of an early warning system (incorporating imported cases, mosquitoes density and climate variability), using existing surveillance datasets will help to control and prevent dengue locally.	NG
AFRICA								
Seidahmed et al., 2012 [44]	Dengue Sudan Port Sudan	Longitudinal study	Spatial and temporal patterns of dengue transmission	Spatial distribution of dengue was irregular in the city. IgM seroprevalence ranged between 3 and 8% among healthy residents and incidence rate was 35 new clinical cases per 10 000 individuals.	Main determinants of dengue outbreaks were increased maritime traffic and weather variation. It should be feasible to carry out timely vector control measures to prevent or reduce dengue transmission in this coastline area.	Not reported.	Further research is needed to define whether there is a true disappearance of *Ae. aegypti* and, if so, how and from where the vector is reintroduced. Study impact of climate and socioeconomic changes on dengue emergence in the Red Sea region.	IG
Malaria								
Authors, Date	VBD Country Region City	Study type	Objectives (outcomes measures)	Results and conclusions	Learning points and recommendations: transmission dynamics, vectorial capacity, co-infection	Main limitations, comments	Future research, public health policy & practice	Funding
AFRICA								
Woyessa et al., 2004 [7]	Malaria Ethiopia	Descriptive	Study malaria transmission in Akaki town, at 2110 m altitude; parasitaemia prevalence; malaria/mosquito species frequency	Parasitaemia in 3.7% of 2136 blood films/3 months (69% vivax, 31% falciparum), none in last month (dry season). *An. arabiensis/An. chrystyi* predominant species (fairly low quantities), suggesting indigenous transmission.	Malaria risk in urbanized African highlands increasing, especially during rains. Possible link to climate change. Long periods of non-transmission reduce population immunity, with risk of recurrent epidemics.	Low case numbers in dry season possibly a result of bednet use/interventions. Malaria transmission low in cities (water pollution) but short-term increase from extra breeding sites during rainy season.	Address vulnerability of highland population of short period malaria transmission and associated epidemics. Apply sustainable and integrated vector control (breeding sites)/case management to prevent epidemics.	U
Ebenezer et al., 2016 [56]	Malaria Nigeria	Descriptive, modelling, spatial	Correlation of *An. gambiae* entomological inoculation rate (EIR) and malaria prevalence and incidence rates in various ecozones	Man-biting rate high (6.9) in mangrove coastal water, vs. fresh/brackish swamp water; *An. gambiae s.s.* infection rate 13% = most efficient and anthropophilic vector (EIR = 70); PR and IR can predict PR-EIR (71%) or IR-EIR (64%).	MBR differs across different eco-vegetational zones of Bayelsa State. Authors propose EIR is a more direct measure of malaria transmission intensity, compared to PR or IR alone.	Methods unclear whether any malaria species or FM only. EIR not suitable for inter-age/population comparisons, due to variable factors such as host biomass.	When assessing efficacy of transmission control measures, both entomological (EIR) and clinical (prevalence and incidence) data need to be considered.	NR
El Sayed et al., 2000 [59]	Malaria Sudan	Descriptive	Transmission in low-income peri-urban Ed dekheinat vs. suburban El manshia, in Khartoum (prevalence rates, mosquito density)	*An. arabiensis* (only vector species) showed higher MBR and indoor density in low vs. high-income areas, especially in rainy season. Parasite screening: FM in high-income areas only, also other species in low-income areas; most FM rates seen in < 15 year age.	Transmission in semi-arid Khartoum unstable, with seasonal pattern and higher risk in low-income peri-urban areas of urban expansion (bringing residential areas closer to cultivated and irrigated land).	Low-income or proximity to agriculture responsible for increased transmission in Ed dekheinat, or interaction? Authors describe major climate, economic, social and political changes to Sudanese capital in recent decades.	Need for improved malaria control to reflect increasing urbanization and changing malaria epidemiology in Africa. Aim for sustained decrease in malaria morbidity and mortality from epidemics.	IG
Ivan et al., 2012 [51]	Malaria and helminth co-infection (HIV infected women > 12 m ART in Rwanda)	Descriptive	Malaria (*Plasmodium* species), helminth or dual infection; cross-sections of peri-urban and rural pregnant women (*n* = 338); prevalence/*OR*	Malaria prevalence lower in peri-urban (12%) vs. rural (30%) areas; also helminth infections (33% vs. 45%; n.s.) high co-infection rates (5% vs. 15%), Nematodes found: *Ascaris* (21%), *Trichuris* (9%) and hookworm (1%);	Possible differential effect of ART regimen type on *P. falciparum*/*Trichuris* co-infection.	Cross-sectional study: no temporal/causality; Study too small to measure true differences, especially potential differential effect of some ART regimens.	Complex immunological profile of co-infection, effect on anaemia (caused by each of the three infections). Malaria/helminth co-infection might facilitate HIV acquisition.	NG, IG
Müller et al., 2001 [50]	Malaria São Tomé	Descriptive	Multiplicity *Plasmodium falciparum* in (FM) protective effect in moderate- transmission West Africa (IR)	61% of Riboque population cross-section PCR positive. The msp-2 genotype multiply-infected had less FM/non-FM over subsequent 3 months.	Multiple *P. falciparum* infections protect against super-infecting parasites; FM prevalence highest in 5–10 year-old children.	Only passive follow-up for febrile illness as possible source of bias (non-attendance, or longterm antimalarial treatment).	Investigate pathophysiology of multiplicity infections: frequently found in asymptomatic infection; protective against superinfection?	NG
Peterson et al., 2009 [49]	Malaria Ethiopia	Descriptive, modelling, spatial	Small-scale spatio-temporal study in 2003 epidemic in Adama (incidence rate ratio)	Kulldorff scan: spatial cluster ≤350 m of breeding site. Other risk factors included: poor housing; proximity to vegetation; ↗temp/rain.	Benefits of identifying temporally stable clusters and risk factors to target cost-effective interventions in transmission hotspots.	Patient may have self-treated or visited other health centres; no data collection in temporary breeding sites.	Small-scale mapping or prediction models to make urban malaria control in Africa more effective.	NR
Sissoko et al., 2015 [48]	Malaria Mali	Descriptive, modelling, spatial	Prevalence of (a)symptomatic malaria/mosquito in 2 areas with different trans-mission/vector distribution	*Anopheles* density/malaria parasitaemia spatial clusters observed in dry season, sometimes associated; but high *Anopheles* density or parasite carriage also found outside the hotspots.	Mosquito density and parasitaemia spatial clusters in small villages (low or mesoendemic malaria transmission), best detected during dry season.	Study areas fairly small and mosquitoes possibly outreaching boundaries. Study done only one month after rainy season.	Efforts to maximize impact of interventions by targeting areas of more intense transmission likely limited by lack of suitability, since high parasite prevalence was detected outside hotspots.	NG, NGO
Ye et al., 2009 [60]	Malaria Burkina Faso	Descriptive, modelling	Predict FM in cohort of children ≤5 years, living in holendemic area (weather/vector data modelling)	Model represented well FM incidence for three ecological settings, including 595 person-years (*n* = 676) over 1 year.	Model predicted seasonal variation increasing vector abundance with increasing temperature 14 days after rainfall; in P. falciparum malaria infection incidence.	Model of parasitological data in children ≤5 years, while entire population contributing to malaria transmission.	Local-scale FM prediction beneficial to guide control; incidence depends on daily vector mortality and human parasite clearance rate, both targets of control measures.	IG, NGO
ASIA								
Dev et al., 2004 [54]	Malaria India	Descriptive	Fever surveys for malaria incidence and risk factors: distance-breeding sites, healthcare facility (IR/RR); vector EIR and weather data	Malaria throughout the year, more in rainy season; mainly FM; incidence higher near streams and foothills/forest areas; lower in areas < 5 km to nearest healthcare facility. EIR and % malaria among fever cases not correlated.	Areas with low-to-moderate EIR could reduce malaria significantly by using campaigns and other tools in combination with GIS methods to target intervention and save costs.	Potential confounding: health centres likely based in plains. Higher *P. falciparum* rates during monsoon (likely due to increased temperatures promoting parasite development in vector).	Health planners and policy makers to consider characteristics of malaria transmission and risk factors in vaccine trials and other, newer approaches for malaria control in this part of the world.	NR
Dhiman et al., 2013 [47]	Malaria India	Descriptive	Impact of altitude on monthly malaria incidence and vector density	↗ temperature coinciding with peak malaria incidence. Malaria transmission window decreased by 1 month with 400 m increase in altitude.	Reduced transmission windows and different vector composition in the highlands.	Malaria incidence based on convenience data from health centres (rather than cross-sectional study of parasitaemia population).	Highland urban areas to be considered vulnerable for malaria transmission, especially due to environmental changes.	NR
Lee et al., 2009 [53]	Malaria Singapore	Descriptive	Malaria trends, epidemiological characteristics, local transmission and control measures (incidence)	1983–2007 incidence ranged from 3 to 11 per 100 000 population, with deaths in 92% due to FM, and 8% vivax malaria, and a sharp decline after 1997. One *P. knowlesi* outbreak.	> 90% of cases imported from other Asian countries; migrants often associated with larger outbreaks in relapsing *P. vivax* cases.	Medical practitioners to highlight risk of malaria to travellers visiting endemic areas and also to consider possibility of simian malaria.	Singapore vulnerable to reintroduction of malaria, requiring high vigilance (e.g. screening; educating on prophylaxis). Consider simian malaria if no travel history.	NR
Zhang et al., 2012 [55]	Malaria China	Descriptive, modelling, spatial	Time series regression of 2006–2010 vivax malaria, vector density, weather variables.	Strong seasonal pattern; peak during 2nd half of year; visual spatial association with average temperature; Tmax/ average humidity (1 m lag); previous month’s incidence.	Increasing *An. sinensis* density likely to contribute only little to malaria incidence in low transmission areas.	Predictive model does not account for human interventions since difficult to measure.	Direct public resources to control infection, rather than vector, when incidence is low. Use surveillance and vary control efforts according to incidence.	NG
Zhao et al., 2013 [52]	Malaria China	Descriptive	Epidemiology of malaria in Ningbo city. Data from case and vector surveillance, local weather and 2008 outbreak analysis	95% of cases were imported vivax malaria from domestic endemic areas, leading to limited local transmission, determined by *An. sinensis* vector density.	Domestic endemic areas are important source for limited, local transmission of vivax malaria. Strengthen monitoring for imported malaria, ensure timely diagnosis/treatment.	No data on floating population (might have significant impact on incidence). Study failed to show rain as precipitating factor for monthly density of female *An. sinensis.*	Future studies to determine impact of floating population on dynamics of local malaria incidence. Focus on timely case detection, diagnosis and treatment.	NR
AMERICAS								
Girod et al., 2011 [58]	Malaria French Guiana	Descriptive	Explore malaria vectors and associated transmission dynamics	*An. darlingi* density high at (though variable between) 3 study locations; only few Plasmodium positive and none at the village with highest rates of human cases.	Variable relationships between malaria incidence, *An. darling* density, rainfall, and nearest river water levels.	Low numbers of infected *An. darling* mosquitoes due to traps located at places away from where transmission was occurring.	Not specifically reported; of note is the strong entomological focus of this work.	NR
Moreno et al., 2007 [57]	Malaria Venezuela	Descriptive/ longitudinal study	Determine anopheline mosquito characteristics and climate factors/ malaria incidence (prevalence)	Transmission throughout the year, with malaria prevalence between 12.5 to 21.4 per 1000 population; *An. darlingii* and *An. marajoara* important vectors, more abundant during rainy season.	Asymptomatic carriers are important reservoir of parasites for persistently high levels of transmission.	Both *Anopheles* species are indoor and outdoor biters but displayed strong exophagic behaviour in villages located in forested areas.	To identify bionomics of *Anopheles* species relevant to malaria transmission in Venezuela for planning and implementing vector control programs.	NG, IG
Other diseases								
Leishmaniasis								
Camargo-Neves, 2001 [64]	Visceral leishmaniasis (VL) Brazil	Spatial	Spatial analysis surveillance tools for VL; Araçatuba, São Paulo, 1998–1999 (incidence)	Heterogeneous transmission: human cases more frequent in areas with high canine rates. Vector dispersion restricted to a few houses.	Benefit of investigating vector distribution and covariates, in a field study based on house sampling.	Limited information on sampling methods; no modelling of vector density.	Need for new spatial analysis tools and redefinition of protocols for control of endemic disease in urban areas.	NR
Salomón et al., 2006 [62]	Tegu-mentary leishmaniasis (TL) Argentina	Outbreak investigation	Distribution of vectors and cases and the risk factors during the 2003 TL outbreak in Bella Vista, Corrientes.	31 cases (25 ± 14 years old), m:f sex ratio = 1.8; compared to matched controls. Clusters in 2 contiguous neighbourhoods; risk higher in peri-urban (96%) than in peripheral (4%) areas.	Urban transmission risk at ecotone-border: changes in human distribution and activities, patches of secondary vegetation, peri-urban streams, rainfall, and river period floods.	Possible over-matching (controls selected in same block of cases) and small sample size: interpret risk factors associated with leishmaniasis infection with caution.	Local-based surveillance system for entomological surveillance, diagnosis, and treatment at sentinel sites. Risk assessment needed for any project involving change in land use at city borders.	NR
Thomaz-Soccol et al., 2009 [63]	Cutaneous leishmaniasis (CL) Brazil	Ecological	Epidemiological and entomological aspects of CL endemicity	61% (*n* = 100) were male rural workers ➔ extradomiciliar transmission. 29% of CL houses had antibody positive dogs. *Lutzomyia intermedia s.l.* most prevalent vector for *Leishmania (V.) braziliensis.*	Diversity of risk factors including domestic dogs, occupational hazards; deforestation to increase agricultural and pastoral areas likely important factor.	Future exploration could emphasize sample size and consider distributional studies.	Further eco-epidemiological/ biogeographical approaches needed to clarify relationships found in the present study, to guide targeted interventions.	NR
Uranw, 2013 [65]	VL Nepal	Outbreak investigation	Investigate possible urban transmission of VL in Dharan town, Eastern Nepal, 2000–2008	115 VL cases (448 controls) strongly clustered in 3/19 (70%) neighbourhoods; independent risk factors include poor housing and low income.	Transmission of anthroponotic VL in urban houses; *P. argentipes* vector and *L. donovani* parasite both identified inside town.	Retrospective nature of study: possible recall bias (> 10 years) during interview in 2009.	Appropriate surveillance and control measures to be adopted not only in rural areas but also in urban areas.	IG
Chagas								
Medrano-Mercado et al., 2008 [67]	Chagas disease Bolivia	Ecological	Demographics of Chagas infections in 5–13 year-old children (*n* = 2218) in Cochabamba, Bolivia, 1995–1999	High seroprevalence in both South (25%) and North (19%) zones, 3× higher *OR* in children aged 10–13 years in NZ: higher triatomine vector infection rate (79% in South and 37% in North), but not density.	Evidence of significant exposure leading to severe disease, already early in life.		*T. cruzi* infection needs to be considered an urban health problem, which is not restricted to rural areas and small villages of Bolivia.	NGO
Salazar et al., 2007 [66]	Chagas disease Mexico	Ecological	*T. cruzi* antibody seroprevalence and associated risk factors in individuals < 18 year in Veracruz, Mexico, 2000–2001	14/1544 samples confirmed positive. Risk factors included seeing reduviid bugs inside house and fissures in roof. Bug infestation index, colonization, and natural infection: 11%, 50%, and 9%, respectively.	Active vertical transmission of infection confirmed, with 0.91% seroprevalence in people <18y, which required attentative follow-up on seroprevalence at population level.		Call for improved housing, vector control measures and surveillance to be put in place.	IG
West Nile virus								
Godsey et al., 2012 [69]	West Nile virus (WNV) USA	Outbreak	Comparison of entomology within and outside WNV outbreak area in Arizona, 2010 (incidence)	Higher *Cx. quinquefasciatus* abundance and proportion of human blood meals detected in 6 outbreak vs. 6 control areas (similar demographics).	*Cx. quinquefasciatus* main vector for transmission, with blood meal host preference and availability increasing risk of WNV infection.	Vector blood meal host preference needs cautious interpretation: represents snapshot during outbreak; also, no bird/mammal census was conducted.	Similar multi-faceted studies during periods of increased WNV transmission to aid development of effective outbreak intervention strategies.	NG
Nielsen et al., 2012 [68]	WNV USA	Spatial modeling	Risk factors during 2006 outbreak in northern California residential community (incidence)	16/1355 (1.2%) pools of *Culex*. WNV positive. Significant spatial-temporal clustering of infected dead birds, positive *Culex* and areas of human cases.	Residential areas had warm night temperatures and WNV-positive *Cx. tarsalis,* likely critical factors during initiation of the outbreak.	Mosquito trapping not conducted throughout the urban landscape.	Need for spatial detection and emergency adult control in locations with WNV activity: interrupt transmission before human infections occur.	U
Chikungunya								
Ho et al., 2011 [71]	Chikungunya Singapore	Outbreak study	Epidemiology of chikungunya establishing itself as endemic disease	2006–2009: 812/1072 (76%) were indigenous cases, imported from India and Malaysia; high risk group: foreign contract workers.	Initially sporadic cases imported into Singapore, then local transmission by *Ae. aegypti* as predominant urban vector.	Introduction of mutant viral strain (A226V) in 2008 resulted in rapid spread by *Ae. albopictus* as primary vector.	Identify and address demographic vulnerability (international travellers and foreign workers). Vigilance needed to detect and respond early.	NG
Rezza et al., 2007 [70]	Chikungunya Italy	Outbreak study	Identify primary source of infection and modes of transmission (risk ratio and attack rate)	Presumed index case was a symptomatic man from India visiting relatives in Italy. Laboratory confirmation was obtained for 175/205 cases (32 PCR only; 135 serology only; 8 both)	High similarity between strains found in Italy and those identified during earlier outbreak in Indian Ocean islands.		Outbreaks in non-tropical areas unexpected; need for preparedness and response to emerging infectious threats in era of globalization.	NR
Yellow fever								
Gould et al., 2008 [73]	Yellow fever (YF) Sudan	Outbreak investi-gation	Confirm cause and further describe outbreak (incidence)	605 cases (163 deaths; CFR 27%); in 177 (29%) illness suggesting YF. Positive IgM-antibodies in 5/18 recently symptomatic, and 4/16 asymptomatic unvaccinated persons. Also chikungunya IgM detected in a few cases, (both ill and asymptomatic).	Serology evidence for both chikungunya and YF during outbreak. Migration and drought likely contributors to outbreak; *Ae. aegypti* most abundant, *Ae. luteocephalus* (important sylvatic YF to both monkeys and humans.	Outbreak diagnosis and response delayed/limited. True YF and chikungunya cases likely underestimated. Limited clinical information and possible recall bias (retrospective study).	Need for timely/adequate response in future outbreaks; improved surveillance for YFV/other arboviruses; Promote YF vaccination in endemic areas and mosquito control for both arboviruses and malaria.	NGO
Vasconcelos et al., 2001 [72]	YF Brazil	Outbreak study	YF outbreaks in Goias and Bahia states, 2000	77 cases reported in 8 Brazilian states with *Haemagogus janthinomys* as main vector. Climate changes with increasing rainfall associated with epidemic and epizootic episodes.	Populations at risk: agricultural workers, tourists, carpenters, fishermen, truck drivers.	Unclear what other factors, besides rainfall, were associated with spread of YF in the area. Further virology studies needed to determine single vs. multiple genotypes.	Need for YF vaccination in all areas recently affected, as well as *Ae. aegypti* control programs for the whole country, to avoid virus re-urbanization.	NR
Ross River virus								
Carver et al., 2008 [74]	Ross River virus (RRV) Australia	Ecological	Relationship between amplifying host (*M. musculus* mouse) abundance and RRV notifications	Mouse index data (proxy for abundance) at Symes Farm matched with RRV incidence in rural townships (postcode) in cereal-growing Walpeup region, Southern Australia.	Mice and RRV notifications significantly related in autumn and autumn+summer when *Culex annulirostris* mosquitoes are abundant.	Cause and effect (mice and RRV cases) not proven: possible other factors required to enable transmission between the two hosts.	More research needed on potential causal relationship (mice and RRV) to justify targeted public health interventions to reduce mice and arboviral disease burden.	NR
Plague								
Pham et al., 2009 [76]	Plague Vietnam	Ecological	Environmental factors and human plague in Vietnam central highland plateau, 1997–2002 (risk ratio)	472 plague cases; 4 main flee and 3 rodent species. Increasing risk during dry and hot months; decreasing rainfall; increasing monthly flea index/rodent density.	Flea index, rodent density, and rainfall could be used as ecological indicators of plague risk in Vietnam’s central highlands plateau.	No wider animal sampling; hence, collected rodents in confirmed cases might not be those causing outbreak. Ecological investigation: no inference to individuals.	Plague outbreaks likely due to multiple ecological factors linked to classical reservoir and vector of bubonic plague; further research warranted.	NG
Rickettsia								
Souza et al., 2015 [75]	Brazilian spotted fever (BSF) Brazil	Case-controlled multivariate regression	Risk factors associated with BSF, 2003–2013, Piracicaba River basin, São Paulo State (*OR*s)	Confirmed vs. discarded BSF cases were associated with increasing age, urban areas, presence of *A. sculptum* ticks, ticks collected from horses, presence of capybaras, and dirty pasture environment.	High incidence areas in São Paulo State reinforce trend of urbanization of BSF in peri-urban peripheries; presence of ticks and capybaras requires future field investigations.	Potential misclassification through use of discarded cases as controls: false negative cases (no timely collection) ➔ *OR* biased towards null hypothesis (no association). .	Control measures needed focusing on elimination of dirty pastures and prevention of human contact with areas of host and vector species, to control transmission of BSF.	NG

DENV: Dengue virus; NR: Not reported; NG: Non Governmental; IG: International Government; U: University; PI: Pharmaceutical Industry; NGO: Non-governmental organization

GIS: Geographic information system; IR: Incidence rate; RR: Risk Ratio; FM: Falciparum malaria; ART: Antiretroviral therapy; NR: Not reported; NG: Non Governmental; IG: International Government; U: University; NGO: Non-governmental organization

**Fig. 3:**
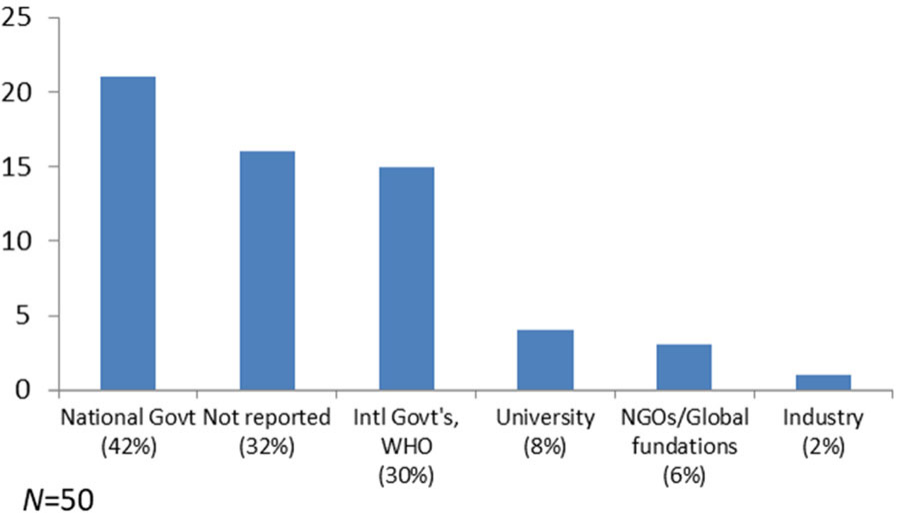
Studies specified according to funding sources, some studies have more than one study source. Funding: not reported, *n =* 16; national government, *n =* 21; university, *n =* 4; international government/WHO, *n =* 15; non-governmental organizations, *n =* 3; pharmaceutical industry, *n =* 1 (some studies had more than one funding source).

Studies were funded mostly through national (*n =* 21; 41%) and international (*n =* 15; 29%) government sources, followed by universities, non-government organizations, and global funding sources (< 10% each). Only one study was funded through pharmaceutical companies, but did not involve clinical trials (hence was not excluded); some studies had several funding sources (Fig. 3).

We transferred information for the 50 included studies into an extraction grid. All studies were descriptive. An overview of study methods employed in each disease group shows that nearly half of the studies on dengue used either spatial (30%) or dynamic (15%) modeling, followed by one third (20% and 13%, respectively) on studies on malaria.. The remainder of malaria research included mostly cross-sectional (*n =* 4; 27%) and cohort studies (*n =* 6; 40%). Studies on other pathogens were mostly outbreak (*n =* 7; 47%) and other case-control (*n =* 3; 20%) studies (Table 3).

**Table 3:** Type of study methods, by disease group

	Dengue *n =* 20	%	Malaria *n =* 15	%	Other *n =* 15	%	All *n =* 50	Total %
Outbreak	2	10%	0	0%	7	47%	9	18%
Case-control (cluster)	2	10%	0	0%	3	20%	5	10%
Cross-sectional	2	10%	4	27%	1	7%	7	14%
Cohort (longitudinal)	3	15%	6	40%	0	0%	9	18%
Analytical - multivariate	6	30%	5	33%	5	33%	16	32%
Ecological (population study)	4	20%	0	0%	5	33%	9	18%
Spatial modelling	6	30%	3	20%	2	13%	11	22%
Time series regression, dynamic modelling	3	15%	2	13%	0	0%	5	10%
Secondary data only	5	25%	4	27%	1	7%	10	20%
Prevalence – Incidence	15	75%	13	87%	13	87%	41	82%
Odds Ratio	2	10%	1	7%	3	20%	6	12%
Risk Ratio or Similar	3	15%	2	13%	3	20%	8	16%

Methods applied in *n =* 50 quantitative descriptive studies in numbers and %, for dengue, malaria and other pathogens (some studies employ more than one different study method)

**Fig. 4:**
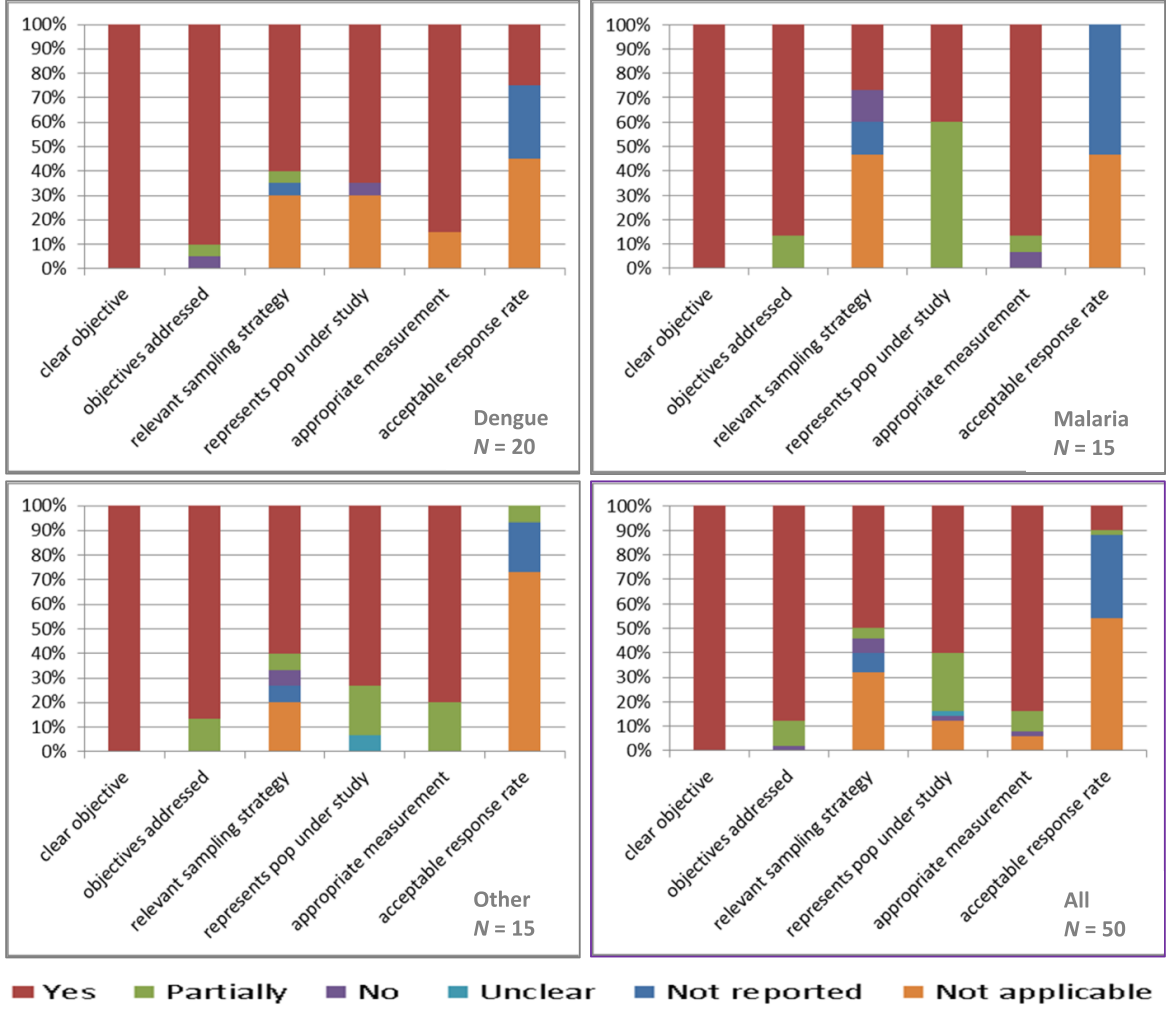
Quality of studies according to modified MMAT tool in numbers (%), for dengue, malaria and other pathogens

We applied the MMAT to evaluate study quality [22]. All studies had clear objectives set out, which were addressed in 90% (*n =* 18) of dengue studies and 87% (*n =* 13) of studies on other VBDs. Relevant sampling strategy for studying human or vector characteristics was present in approximately 65% of studies on dengue and other pathogens, but to a lesser degree (*n =* 4, 27%) in malaria work. Representation of the population under study was also better addressed in studies on dengue and other pathogens (around 70%) than in malaria studies (*n =* 6; 40%). Appropriate measurement was captured well in both dengue and malaria studies (*n =* 17, 85% and, *n =* 13; 87%, respectively). Response rate (where appropriate) was clearly reported only in about 25% of dengue studies and even less in other work (Fig. 4). Given the absence of any intervention studies, the TIDieR tool was only applicable to very limited aspects of the included studies. Due to the limited added benefit, it was therefore agreed to not consider TIDieR further in the extraction.

### Description of findings of the scoping review

#### Dengue transmission dynamics and vectorial capacity studies

Dengue research was identified mostly in the Americas and Asia. The majority of the studies (*n =* 17) evaluated the relationship between the incidence of dengue cases and vector density in endemic areas, and/or mobility of the human population. A large number of studies also addressed asymptomatic infection as a factor related to the spread of dengue virus infection, the climatic variation in relation to vector abundance, and the role of socioeconomic conditions. The role of imported dengue fever cases in triggering outbreaks in non-endemic cities was highlighted [24]. Human movement due to economic development and/or tourism was considered a determinant for the spread of dengue infection [24–26].

#### Incidence of dengue cases or dengue virus infection

The heterogeneity of dengue transmission in inner cities seemed to be a common feature of the studies. Population immunity and asymptomatic infection play an important role in dengue transmission dynamics, resulting in higher incidence of dengue infection in previously lower prevalence areas [25]. Also, the intensity of transmission in highly urbanized settings may not be perceived as an epidemic due to asymptomatic infection, suggesting the occurrence of a “silent epidemic”, as shown in Salvador city, Brazil (2008–2009) [25]. Another prospective study demonstrated that dengue infection in the community was mainly spread by asymptomatic adults [27]. In concordance with these studies, asymptomatic dengue cases were also a potential source of subsequent outbreaks, as seen in four cities of Valle del Cauca, Colombia [26]. A study conducted in Ho Chi Minh city, Vietnam, provided evidence for some household dengue risk clustering, but on a short temporal scale rather than as sustained chains of localized transmission [28]. These findings are important for surveillance and control strategies [25–27].

#### Mobility of human populations as a source of outbreaks and/or disease persistence

The study conducted in Mexico City (2011–2012) pointed out that, besides asymptomatic individuals, human mobility is another important factor in the spread of dengue infections in urban settings [27]. The spread of dengue by imported cases has been reported in some countries, such as Argentina during the 2009 outbreak [29], as well as China in the outbreaks in the outskirts of Cixi [30] and Dongguan cities [31] and near Guangzhou Baiyun International Airport (2006–2012) [24]. The reports from China highlighted the role of immigrants from Southeast Asia as the source of localized outbreaks in this region.

#### Vectorial capacity and incidence of dengue infection or disease

There was conflicting evidence around indicators of vector abundance and incidence of dengue infection/disease. Positive associations between high vector density and high dengue incidence were reported in different settings, such as San Juan, Puerto Rico [32], Cuba [33], Argentina [29], Brazil [34, 35] and Bangladesh [36]. In contrast, other studies showed inverse relationships between entomologic indices and dengue incidence [26, 37–39]. It has been well established that water storage containers and discarded tires are important mosquito habitats in many countries, which was also reinforced by studies in Puerto Rico [32] and French Guiana [40].

#### Weather and climate variability and vector proliferation

Studies conducted in different regions evaluated the role of weather on the proliferation of *Aedes aegypti,* dengue incidence, and the seasonality of the disease [30, 32, 39, 41–43]. Some ecological studies observed a positive association of temperature and rainfall with variations in the *Aedes* mosquito population [32, 38, 39]. Some studies conducted in the Americas showed a positive correlation of rainfall and temperature with dengue incidence [32, 41], while others in Asia found negative or no associations between these meteorological variables and dengue incidence [30, 42, 43]. In an interdisciplinary study (2010–2011) conducted in São Paulo, Brazil, using geographic information systems, more dengue cases were clustered in areas of land surface temperature above 32 °C than in areas characterized as low socioeconomic, high population density areas, or slum-like areas [39]. That study’s findings were interpreted as suggesting that the land surface temperature of the inner city was a better predictor for dengue incidence than were other factors such as population density or socioeconomic indicators. Therefore, the influence of higher temperatures in small urban areas in São Paulo, known as urban heat islands, was correlated with high-risk areas of dengue transmission during this period (2010–2011) [39].

#### Other social and environmental factors influencing or associated with the complexity of urban settings

Other social and environmental factors associated with disease incidence and vector abundance include living and working conditions, human behaviour, urban infrastructure, and water and sanitation, which includes water storage conditions and housing quality [30, 32, 37, 40]. In Sudan, a study conducted in the neighbourhoods of the city of Port Sudan (2008–2009) [44], observed that dengue incidence was heterogeneously distributed and higher entomological density indices were found in lower- and middle-class neighbourhoods. In that setting, climate variability, maritime traffic, and socioeconomic conditions were suggested as being the main drivers of dengue outbreaks in the past decade, although further research will be required to study the impact of long-term climate change on dengue emergence in that region [44]. In Rio de Janeiro, Brazil, spatial analysis showed a positive association between social inequalities (Gini indices) and the container (Breteau) index for *Aedes albopictus* [41]. A serosurvey conducted in the US-Mexican border area found an abundance of vectors in US cities, but higher dengue incidence in neighbouring cities in Mexico. This lead the authors to conclude that population living conditions (low air conditioning rates, small living spaces, high numbers of occupants) were the main determinants of dengue transmission in that region, indicated by seroprevalence in each population [37].

### Malaria transmission dynamics and vectorial capacity studies

The malaria research retrieved was mostly conducted in the African region, where yearly estimates indicate to be the highest burden of malaria cases (191 million cases in WHO African Region vs. 21 million in other parts of the world) and deaths; and the highest proportion of *Pl. falciparum* (vs. other species) worldwide in 2016 [5]. At the same time there is the lowest level of health expenditure in this compared to other regions [45], further aggravating the impact of the disease on populations and economies.

#### Incidence of malaria cases or infection

There has been controversy about a potential expansion of malaria from rural areas into cities. Research evidence suggests increased malaria risk to urban dwellers, and transmission in urban and periurban setting [46]. In the studies selected in our review, increasing transmission risk was described as part of climatic changes affecting urban areas in the highlands of Ethiopia, and India, respectively [7, 47]. A study using small-scale temporal-spatial scanning identified “hotspots” of high *Anopheles* density and parasite carriage in two villages in Mali. However, transmission was also found to be high outside these hotspots, casting doubt on the effect of targeted control interventions in hotspots [48]. A transmission hotspot detected within 350 m of a large *Anopheles* breeding site during the 2003 outbreak in Ethiopia highlighted the potential of targeted vector control measures to reduce cases [49].

With regards to the occurrence of co-infections, Müller et al. [50] found a protective effect in children infected by multiple, genetically different *Pl. falciparum* malaria parasite co-infections. Further, there was a protective effect against episodes of febrile illness during a three-month subsequent (passive) follow-up. The group found an 0.84 (95% *CI*: 0.71–0.99) hazard associated with each additional *Pl. falciparum* genotype detected at baseline and called for more research into this phenomenon, in particular the effects on the host in chronic infections [50]. Another group studied malaria and helminth dual infections among pregnant women with HIV infections on treatment for > 1 year. The study design was merely descriptive, showing co-infection rates to be higher in women living in urban areas than among those in rural areas [51]. The authors hypothesized regarding potential aggravating effects of co-infection on anemia, which is a consequence of each of these three types of infection (malaria, helminths, HIV).

#### Mobility of human populations as a source of outbreaks and/or disease persistence

Zhao et al. [52] found most malaria cases in Ningbo City, China, to be caused by *Pl. vivax* imported from domestic endemic areas, leading to local transmission through *Anopheles sinensis.* The authors suggested that more research was needed on the role of floating populations in local malaria transmission [52]. Migration and travel were also identified as important risk factors for malaria re-introduction in Singapore, evidenced by an analysis of 25-year reporting data. The authors advocated for screening, education, and good case management. Finally, they suggested that also simian *Plasmodium knowlesi* malaria would need to be considered as a possible source of fever in their study population [53].

#### Vectorial capacity and the incidence of malaria infection or disease

Several studies demonstrated changes in vector composition according to geography and season to explain variations in transmission [47, 52, 54, 55]. There was diversity among studies on the correlation between entomological parameters and human malaria. The EIR as indicator for transmission was found to correlate with clinical prevalence and incidence data in Nigeria [56], similar to *Anopheles* density and malaria cases in Ningbo, China [52]. An important factor for malaria transmission in forested villages in Venezuela was the presence of mosquito species displaying predominantly exophagic (outdoor) biting behaviour [57].

In contrast, no correlation was found between EIR and percentage of malaria among reported fever cases in India [54], nor between *Anopheles* density and human case rates in French Guiana, and the authors argue the mosquito traps might have been located in non-transmission areas [58].

#### Weather and climate variability and vector proliferation

Increased transmission during the rainy season has been observed in Africa and Asia [7, 54, 59]. More specifically, increasing temperature and rainfall were followed by higher vector prevalence transmission models based on four different areas of Burkina Faso, with peak vector prevalence occurring 2 weeks after peak rainfall [60]. In northwest China, monthly *An. sinensis* vector density (relevant for *Pl. vivax* malaria transmission) was strongly correlated not only with temperature (*R* = 0.958, *P* < 0.001), but also with humidity and rainfall (*R* = 0.746, *P* = 0.005; and *R* = 0.725, *P* = 0.008, respectively) [52]. Furthermore, increases in average maximum and minimum temperatures (at 1 month lag) and rainfall (10-week lag) in Ethiopia had malaria incidence risk ratios of 1.4 (for maximum temperature), 1.3 (for minimum temperature), and 1.0 (for rainfall) [49]. Zhang et al. (2012) showed malaria epidemiology in China to have strong spatial associations with average temperature. They proposed optimizing case management rather than vector control for low-transmission areas [55].

#### Other environmental factors, geography, and complexity of urban setting influencing malaria infection or disease

Peterson et al. (2009) identified not only proximity to a large *Anopheles* breeding site as a source of increased transmission, but also poor housing as a further important risk factor (malaria incidence risk ratio = 2.0) in Adama, Ethiopia [49]. Similar observations were made in semi-arid neighbouring Sudan, where transmission was higher in rapidly expanding peri-urban low-income areas than in suburban higher-income areas of Khartoum [59].

Certain ecological areas show higher malaria transmission than others. In Nigeria, transmission rates (MBR and EIR) were higher near mangrove coastal water than in areas of fresh or brackish water [56]. In Ethiopia, proximity to vegetation and to agricultural sites showed higher vector density and more cases [49, 54, 59].

Further, changes to ecology and climate were considered to be causing increasing malaria transmission in urbanized highland areas of Africa and Asia [7, 47]. Capable *Anopheles* vectors and short-term malaria transmission were observed during the rainy season (due to short-term extra breeding sites) in Ethiopia, where low population immunity between seasons causes epidemics [7]. Comparison of three villages at different altitudes in India showed both reduced *Anopheles* abundance and transmission windows for human cases with increasing altitude [47]. Geographic expansion of mosquito vectors has been described as one of the possible effects of climate change [61].

Summarizing key insights from respective groups of authors, there was an expression of need to address the increased risk of transmission in vulnerable highland areas [7, 47] and in spaces where specific risk factors are present, including proximity to breeding sites, poor housing [49], low income [49], and floating populations [52, 53]. They recommended better ways to monitor and address risk factors via spatial studies and forecast models that include entomological parameters and meteorological factors. Further, the significance of asymptomatic infections was expressed in reports on parasitaemia and floating populations, identifying the need to detect and treat such cases to prevent transmission [52, 53, 59]. Finally, the complexity and potential impact of co-infections on the host have been recognised [50, 51].

### Other diseases

Four studies focused on leishmaniasis: two on cutaneous leishmaniasis (in Argentina and Brazil) [62, 63] and two on visceral leishmaniasis (in Brazil and Nepal) [64, 65]. Agricultural male workers were identified as a risk group; further associated factors included peri-urban living environment, low socioeconomic status, poor housing, and domestic dogs. All reports demonstrated the need for improved surveillance and control measures, to reduce infection risk both in urban and peri-urban areas, with specific focus on dog populations.

Chagas disease was detected in young individuals in Mexico (1% of people aged < 18 years) [66] and Bolivia (> 20% of school children aged 5–13 years). Poor housing and high infection rates among transmitting vectors were identified as important risk factors; the authors suggested prioritizing detection and control programs in these urban areas [67].

West Nile virus (WNV) is a VBD in which bird populations such as corvids serve both as important reservoir and amplifiers, whereas migratory birds are involved in global transmission [68]. Researchers in the United States (US) correlated mosquito and local bird population WNV status with human incidence. They identified *Culex* mosquitoes as important vectors in the Arizona outbreak where their abundance and (bird) host preference increased the risk of human transmission, and compared to control sites in the metropolitan area of Phoenix (US) [69]. A spatial study identified significant clustering of infected dead birds and positive *Culex* mosquitoes near human cases occurring in residential areas of California [68]; early detection was proposed as a key to reducing the risk of outbreaks.

#### Role of tourism, migration and occupational exposure on transmission of infection

Two studies reviewed chikungunya occurrence, one in Italy following virus introduction by a symptomatic individual visiting from India [70] and the other in Singapore, where recent virus mutation allowed the infection to be effectively transmitted by urban *Aedes albopictus* mosquitoes [71]. Both research groups highlighted the role of migrants in the spread of disease and the need for effective disease surveillance to prevent outbreaks.

In Brazil, researchers identified a yellow fever transmission link to tourism and occupational exposure (agricultural workers, carpenters, fishermen, truck drivers) and to *Haemagogus janthinomys* as the main mosquito vector [72]. In Sudan, drought, migration, and the lack of diagnostic capabilities or adequate response contributed to a yellow fever outbreak where there was concurrent transmission of chikungunya [73].

#### Influence of disease ecology on transmission

A field survey found seasonal abundance of amplifying mouse populations to increase Ross River virus (RRV) transmission in Australia, combined with the presence of the *Culex annulirostris* vector. The authors proposed more specific research on the causal relationship between mice and RRV, along with possible interventions to control the disease [74].

Bacterial diseases studied included tick-transmitted Brazilian spotted fever (BSF) in Brazil [75] and human bubonic plague in the Vietnam Central Highland plateau [76], for which multiple ecological factors were identified, and the authors proposed using rodent density and rainfall as ecological risk indicators.

## Discussion

Dengue and malaria studies constituted the largest groups of published research in our review—dengue predominantly in Asia and the Americas, and malaria in Africa. Dengue has the highest burden and vectors capable of transmitting in urban and peri-urban areas of these regions. The urbanization of the population in Africa has also reflected in malaria transmission that can be currently considered an urban problem [77]. Despite being different pathogens (protozoa vs. virus), both VBDs, despite spread by different mosquito species can be framed with regards to the importance ofurban heat islands and eco-zones, human habitat (proximity to breeding sites), host behaviour and mobility, the role of asymptomatic infections, and association with increased temperature and rainfall (albeit more evidence is required on the associations between climate variability and dengue incidence to explain the discrepancies in recent studies) promoting vector abundance and associated disease incidence. Since the beginning of 1900 malaria research employed the theory of Ross-MacDonald for the dynamics and control of mosquito-transmitted pathogens. This transmission model has now been adopted for dengue research, which has become more intensified in recent years as the disease is becoming a global problem [19].

Dengue transmission and vectorial capacity have been studied mainly using the conventional framework of interaction between human and mosquito populations. A bulletin of the World Health Organization highlighted the importance of increasing residents’ knowledge regarding dengue transmission, which was associated with a measurably lower mosquito reproduction in the respective areas [78]. This was presented as an example of how broader public health efforts (beyond larvicide and focal spraying) can contribute to effective vector control [78]. There is a lack of translational research and a need to combine multiple knowledge areas involving urban planners, travel and border agencies, transport authorities, environmentalists [79]. Such integration would be an useful approach to better understand and respond to the complexity of dengue dynamics in urban settings. Only a few studies addressed this using information on previous dengue serotype immune status to understand disease spread and persistence.

None of the selected studies assessed the co-circulation of VBDs transmitted by the same vector, such as dengue, chikungunya and Zika, which coexist in many regions across the globe [11]. A syndromic approach focusing on patients’ main symptoms, such as fever and rash (equally common symptoms for dengue, chikungunya, Mayaro, Zika, etc.), rather than only on isolated pathogens, might help to adapt VBD research more effectively to the clinical-epidemiological reality. Combining such an approach with broad diagnostics (e.g. testing for a panel of common vector-transmitted parasites, viruses, and bacteria) would allow easy detection of and response to co-circulating vectors, including newly emerging pathogens. This is particularly true for a coordinated international response to new pathogen introductions or epidemics, such as Zika in the America. Harmonization of syndrome-based protocols would increase the effectiveness of such efforts.

Similarly, malaria has been studied largely in conventional frameworks. In some studies, quality was very basic: questionable sampling techniques (convenience sampling), no indication of the proportion of non-responders, offering only passive rather than active follow-up, location of mosquito traps not matching with areas of human transmission—all of which created risks of bias. Also, most studies were descriptive (i.e., using measures of occurrence) rather than providing robust figures of transmission risk (measures of effect). Further, a multidisciplinary approach, as suggested above, could have provided essential insights into the role of asymptomatic infections, especially among floating populations.

Discussing other infections than dengue and malaria, we detected recommendations on specific surveillance and control measures that were included in most studies. For example, the need for entomological surveillance and control in detecting risk areas for Leishmaniasis [62, 63, 65], Chagas’ disease [66, 67], and arboviruses (West Nile Virus [68, 69], Chikungunya [71]), and plague [76]. Also, the need of targeted surveillance and interventions focusing on important animal reservoirs for Leishmaniasis (dog population) [63, 64], West Nile Virus (clustering of dead birds) [68], Ross River Virus (abundance of house mice), and plague /BSF (rodents) [75, 76]. The importance of increasing such measures specifically in urban and periurban areas was highlighted in relation to Leishmaniasis [62, 64, 65], Chagas’disease [67], West Nile virus [68] and BSF [75]. In addition, enhancing vigilance around migration and travel are needed to reduce risk for spreading of Chikungunya [70, 71] and Yellow Fever [72, 73]. For the latter, the importance of vaccination programs was mentioned [72, 73]. Considering that two thirds of the studies were funded by government sources (national or international) the an integrated approach including human and animal health, and entomology should be reinforced. The Joint external evaluation tool by the World Health Organisation as part of Global Health Security is an example for multi-sector and multi-disciplinary effort. This agenda considers multiple hazards, including detection and control of priority epidemic diseases, border surveillance, using an integrated ‘One Health’ approach including human, animal and environmental health [80].

This scoping review has some limitations.

Performing a detailed data extraction on all 205 papers was considered as unfeasible by the consortium. Therefore, an additional step to include only studies that covered at least two of the key concepts (i.e. “transmission dynamics and vectorial capacity” and, “transmission dynamics and co-infection”) limited the number of papers. An additional benefit of this approach was a more comprehensive picture that combined at least two components of infectious diseases in urban areas. At the same time, we acknowledge limitations arising from this final step, which may have excluded important papers reporting only one key concept. That way, classical modelling studies, (particularly those dealing solely with mathematical models (for prediction of outbreaks, spread of infection, and/or long term sustainability of transmission) may have been missed [17, 18, 81].

Further limitations relate to quality and comparability of selected work. Only a few studies went beyond description, as shown by MMAT evaluation of the quality of studies. Clear objectives were set out in all studies and were addressed to a fairly large extent. However, concerns about study quality arose regarding 10–20% of studies, which did not report the relevant sampling strategy. Compared to the studies on other pathogens, the malaria studies were less representative of true population (therefore producing less generalizable results), due to their designs, which were mostly smaller-volume cross-sectional and cohort studies.

Also, there was no attempt to stratify by population size. Stratification would have allowed to identify challenges specific to highly populated urban areas as opposed to smaller urban areas like villages. Dengue is a VBD amplified by humans (rather than other non-human hosts) which contributes to large scale transmission in cities. In contrast, transmission dynamics for a number of other pathogens included (e.g. Leishmaniasis, West Nile Virus, Yellow Fever, Ross River virus and plague) rely on non-human host species, such non-human primates, dogs, rodents or birds. Presence of those species will depend on different types of urban and peri-urban environments and other factors. The same applies for the type of insect vectors implicated. From this perspective, further work classifying between different urban environments will be useful.

The difference in methods used by studies is one factor limiting the comparability, particularly on study that combined weather and entomological data to predict VBD incidence [24, 31, 42, 60]. Differences also arose in the researchers’ selections of the most appropriate ways to control for factors such as seasonality and non-linearity of weather dependence, as pointed out in a technical paper on temporal modeling research [82]. Of note is that, to our knowledge, there are currently no international standards to advise on the most appropriate modeling approach for real-time prediction to inform public health practice.

Finally, another limiting factor was that only two studies reported on co-infections, and both of those addressed malaria. This is concerning in view of how little is known about this phenomenon, the immunological mechanisms involved, and what it means for clinical outcomes; even less is known about transmission dynamics.

**Table:** 

**Box 1** The main implications for future research and public health policy and/or practice	
Knowledge gaps and priority needs for future research	
1. Assess the magnitude of asymptomatic dengue infection at population level (surveillance of symptomatic dengue cases is insufficient to evaluate the persistence of infection).	
2. Improve indoor and outdoor vector density parameters for more accurate modeling of transmission.	
3. More studies are needed on climate and other environmental (e.g. land surface temperature) changes and their effect on vector proliferation and dengue transmission.	
4. The impacts of human mobility within and between cities and countries should be prioritized in future research.	
5. Enhance research and seek scientific consensus on the benefit of simple, ready-to use forecasting tools to predict human VBD risk (using entomological, meteorological, and other parameters).	
6. Promote research on co-infections with different pathogens, on immunology mechanisms and their effect on clinical outcomes and onward transmission, and on means of effective diagnosis and treatment.	
Implications for public health policy and/or practice	
1. In dengue-endemic areas, monitoring areas of low transmission may be necessary to prevent spread of infection.	
2. Surveillance and control strategies focused on index cases should be timely to avoid time lag between outbreak onset and response.	
3. Asymptomatic individuals contribute to persistence of dengue and malaria transmission, reinforcing the need for population screening (e.g. biological marker laboratory testing blood banks, sentinel sites), in low and high seasonality.	
4. Need to assess multiple data sources regarding symptomatic and asymptomatic cases.	
5. Surveillance and control strategies focused on index cases should be timely to stop transmission.	
6. Greater efforts must be made to translate knowledge about VBD transmission into practice.	
7. Employ scientifically agreed-upon ready-to use forecast models to predict human VBD risk based on entomological and meteorological parameters.	
8. Increased rainfall and humidity, especially during the rainy season, affects VBD transmission; authorities need to collaborate to heighten vigilance and control measures.	
9. Poor housing, low-income neighbourhoods are high-risk areas for VBD transmission; they should be focus of affordable and sustainable vector control measures in homes, workplaces and schools, to lower transmission over the long term.	
10. Certain occupational groups have higher exposure to VBDs; labour and agricultural authorities must invest in efforts to increase awareness and safety in relation to specific disease risks.	
11. Transport authorities and border agencies need to screen floating populations at risk of infection.	
12. Using a syndromic approach instead of the classic single-disease surveillance would allow timely response to the introduction of new pathogens or early outbreak detection.	
13. Harmonization of protocols are needed to facilitate a coordinated international effort to control disease threats of national/international importance. National government and academic institutions to promote an integrated multi-disciplinary approach (human and animal health, vector control), focusing on detection and control of priority epidemic diseases, border surveillance.	

## Conclusions

The present review identified significant knowledge gaps in several areas, ranging from the role of asymptomatic individuals to the effects of co-infection and various host characteristics, climate, and other environmental and socioeconomic factors on VBD transmission in urban areas. There is much more to know about transmission risk in the homes and workplaces of increasingly dynamic and mobile populations.

The lack of studies on co-infection is hampering the monitoring of infections transmitted by the same vector. A broad, syndromic approach including pathogen panels would allow more flexibility in detecting new and co-circulating pathogens and in applying more effective control. It would be useful to combine this with harmonized protocols and to define sentinel areas in order to enable a well-coordinated international response where needed. Due to the complexity of VBD transmission, funding for translational research is especially recommended.

## Additional files

Additional file 1: Multilingual abstracts in the six official working languages of the United Nations. (PDF 390 kb)

Additional file 2: Literature search: Associated keywords, MESH and search terms. (DOCX 25 kb)

Additional file 3: Final reference selection by country. (DOCX 15 kb)

## Abbreviations

ART: Antiretroviral therapy
BSF: Brazilian spotted fever
CAR: Conditional Autoregressive
CFR: Case fatality rate
CL: Cutaneous Leishmaniasis
EIR: entomological inoculation rate
FM: Falciparum malaria
IG: International Government
IR: Incidence rate
LST: Land Surface Temperature
MBR: Man biting rate
MMAT: Mixed Methods Appraisal Tool
n.s.: Not significant (statistically)
NG: Non Governmental
NGO: Non-governmental organization
NR: Not reported
OR: Odds ratio
PCR: Polymerase Chain Reaction
PI: Pharmaceutical Industry
PR: Prevalence rate
RF: Risk factors
RR: Risk Ratio
RRV: Ross River virus
TDR: Tropical Diseases Research and Training
TIDieR: Template for Intervention Description and Replication
TL: Tegumentary Leishmaniasis
U: University
UHI: Urban Heat Island
VBDs: Vector-borne diseases
VL: Visceral Leishmaniasis
WHO: World Health Organization
WNV: West Nile virus
YF: Yellow Fever
YFV: Yellow Fever Virus
