# Supplementary material for: Scoping review on vector-borne diseases in urban areas: transmission dynamics, vectorial capacity and co-infection
Source: Infect Dis Poverty. 2018 Sep 3;7:90. doi: 10.1186/s40249-018-0475-7 (PMC6120094; doi:10.1186/s40249-018-0475-7)
Supplement: Supplementary file 1 — Multilingual abstracts in the six official working languages of the United Nations. (PDF 390 kb) [file 40249_2018_475_MOESM1_ESM.pdf]

Translation of the abstract into the six official working languages of the United Nations

مراجعة استطلاعية للامراض المنقولة بالنواقل في المناطق الحضرية: ديناميات النقل ، والقدرة القيادية والعدوى المشتركة

ماركوس ايدر ، فاني كورتيس ، نوميا تيكسيرا دي سيكوريا فيلها ، جيوفاني فينس اراخو دي فرنسا ، ستيفاني گروته ، سينثيا براغا ، فاليري ريدي ، سيلينا ماريا تورتشي مارتيللي

نبذة مختصرة

معلومات أساسية: وتؤثر ديناميات النقل ، وقدرات التصميم ، والتهابات مشتركة تاثيرا كبيرا على الامراض المنقولة بالنواقل التي تصيب سكان المدن والضواحي. ويمكن لاستعراض العوامل الرئيسية ان يوفر نظره ثاقبه على مجالات البحوث ذات الاولويه وان يقدم اقتراحات للتدخلات المحتملة.

الجزء الرئيسي: من خلال مراجعة استطلاعية، لتحديد الثغرات المعرفية في ديناميات الإرسال ، وقدرة التصميم ، والعدوى المشتركة فيما يتعلق بالمناطق الحضرية. وجري البحث في المنشورات غير الرسمية واستعراض الأقران الذي نشر بين عامي 2000 و 2016. قمنا بفرز الملخصات والنصوص الكاملة لاختيار الدراسات. وباستخدام شبكه استخراج ،استرجعنا البيانات العامة ،والنتائج ،والدروس المستفادة والتوصيات ، وسبل البحث في المستقبل ،والآثار المترتبة على الممارسة. وقمنا بتصنيف الدراسات بواسطة ناقل الامراض المنقولة والبلد/القارة وحددنا الثغرات المعرفية ذات الصلة. ومن 773 ماده اختيرت للفحص بالنص الكامل ، أدرجت 50 في الاستعراض: 23 على أساس البحوث في الأمريكتين ، و 15 في اسيا ، و 10 في افريقيا ، وواحد في كل من أوروبا وأستراليا. وكانت أكبر مجموعه من الادله المتعلقة بناقل الأمراض المنقولة بعلم الاوبئه في المناطق الحضرية تتعلق بحمى الدنك والملاريا. ومن بين الفيروسات الأخرى المغطية بفيروس المشتل شيكونغونيا وغرب النيل ،والامراض الطفيلية الأخرى مثل داء الليشمانيات وداء المثقبيات ، والبكتيريا البكتيرية والطاعون. معظم المقالات المسترجعة في مراجعتنا تجمع بين ديناميات الإرسال والقدرة القيادية؛ فقط اثنين من ديناميات الإرسال المشتركة والعدوى المشتركة. وحددت المراجعة ثغرات معرفيه كبيره بشأن دور الافراد الذين لا اعراض لهم ، وأثار العدوى المشتركة وغيرها من العوامل المضيفة، وآثار العوامل المناخية والبيئية والاجتماعية-الاقتصادية على انتقال المرض في المناطق الحضرية. وشملت القيود المفاضلة بين تقليص استراتيجية البحث (المفقودة في دراسات النمذجة الكلاسيكية) ، ونقص الدراسات عن الإصابات المشتركة، وكون معظم الدراسات وصفيه فقط ، وقله منها تقدم توصيات ملموسه في مجال الصحة العامة. ويلزم إجراء مزيد من البحوث بشأن مخاطر الانتقال في المنازل وأماكن العمل، نظرا لتزايد دينامية السكان وتنقلهم. ويتيح استخدام وسائط الاعلام الاجتماعية إمكانيه كبيره للكشف عن أنماط الانتقال الأوسع نطاقا والتحكم فيها. ويعيق الافتقار إلى الدراسات المتعلقة بالعدوى المشتركة رصد الإصابات التي ينقلها الناقل نفسه.

الاستنتاجات: ومن الاستراتيجيات الرئيسية التي حددت السياسات وممارسات الصحة العامة تعزيز التحكم ومراقبة ناقل الأمراض المنقولة، ولا سيما في حالات الاعراض والسكان المتنقلين، فضلا عن استخدام أدوات الإنذار المبكر للتنبؤ بزيادة انتقال العدوى.

Translated from English version into Arabic by Free bird, proofread by GHANIA Khalifa, through

TRANSLATORS
WITHOUT BORDERS

城市地区媒传疾病的传播动力学、媒介能量与合并感染：堪域综述

Marcus Eder, Fanny Cortes, Noêmia Teixeira de Siqueira Filha, Giovanny Vinícius Araújo de França, Stéphanie Degroote, Cynthia Braga, Valéry Ridde, Celina Maria Turchi Martelli

摘要

引言：传播动力学、媒介能量和合并感染对城市和郊区人群的媒传疾病产生重大影响。对关键因素进行回顾综述可深入了解研究重点领域，并提供可能的干预建议。

主要内容：通过对 2000–2016 年间出版的经同行评议的文献和灰色文献进行检索，并进行堪域综述以确定城市地区VBDs传播动力学、媒介能量和合并感染的知识差距。我们通过浏览摘要和全文筛选文献。通过提取网格，

检索了一般数据、结果、经验教训和建议、未来的研究途径和实践意义。根据 VBD 的疾病种类和研究地区（国家/洲）对文献进行分类，确定相关的知识差距。对 773 篇文章进行全文筛选，纳入其中 50 篇：23 篇来自美洲，15 篇来自亚洲，10 篇来自非洲，欧洲和澳大利亚各 1 篇。城市地区 VBD 流行病学方面的研究主要来自登革热和疟疾。此外，还有其他病毒病（如基孔肯雅病和西尼罗河病）和寄生虫病（如利什曼病和锥虫病），以及细菌性立克次体病和鼠疫。本次综述所检索到的大多数文章同时涉及传播动力学和媒介能量，只有两篇同时提及传播动力学和合并感染。研究发现，无症状个体的作用、合并感染的影响和其他宿主因素的影响以及气候、环境和社会经济因素对城市地区 VBD 传播影响方面的研究存在显著的知识差距。本文的局限性包括缩小搜索策略的范围(遗漏了经典的模型研究)，缺乏合并感染的研究文献，大多数仅为描述性研究，极少数的研究提供了具体的公共卫生建议。由于人群越来越具有活动性和流动性，因此需要对家庭和工作场所的传播风险进行更多的研究。社交媒体的使用为检测和控制更广泛的传播模式提供了巨大潜力。对合并感染研究的不足妨碍了监测同一病媒所传播的疾病。

结论：加强 VBD 监测和控制，以及使用预警工具预测不断增加的疾病传播，是公共卫生政策和实践确定的关键战略，特别是在无症状病例和流动人群中。

Translated from English version into Chinese by Xin-Yu Feng, edited by Pin Yang

CHINESE CENTER FOR DISEASE CONTROL AND PREVENTION
NATIONAL INSTITUTE OF PARASITIC DISEASES

Examen de la portée des maladies à transmission vectorielle dans les zones urbaines : dynamique de la transmission, capacité vectorielle et co-infection

Marcus Eder, Fanny Cortes, Noêmia Teixeira de Siqueira Filha, Giovanny Vinícius Araújo de França, Stéphanie Degroote, Cynthia Braga, Valéry Ridde, Celina Maria Turchi Martelli

Résumé

Contexte: La dynamique de la transmission, la capacité vectorielle et les co-infections ont un impact important sur les maladies à transmission vectorielle (VBDs) qui affectent les populations urbaines et suburbaines. Une étude des facteurs clés permet d'offrir un aperçu des domaines de recherche principaux ainsi que des suggestions d'interventions possibles.

Corps principal: identifier, à travers un examen de la portée, les lacunes de connaissances sur la dynamique de transmission, la capacité vectorielle et les co-infections relatives aux maladies vectorielles dans les zones urbaines. De la documentation évaluée par des pairs et de la littérature grise publiée entre 2000 et 2016 ont été examinées. Nous avons examiné des extraits et des textes entiers afin de sélectionner les études pertinentes. À l'aide d'une grille d'extraction, nous avons récolté les données générales, les résultats, les leçons apprises et recommandations, les futures pistes de recherche et les incidences pratiques. Nous avons classé les études par maladie vectorielle et pays/continent et identifié les lacunes de connaissances appropriées. Sur 773 articles sélectionnés pour un examen du texte intégral, 50 ont été inclus dans cette étude : 23 basés sur des recherches en Amérique, 15 en Asie, 10 en Afrique, une en Europe et une en Australie. La majeure partie des données sur l'épidémiologie des maladies vectorielles dans les zones urbaines concernent la dengue et la malaria. Parmi les autres arbovirus abordés, on retrouve le chikungunya et le virus du Nil occidental, d'autres maladies parasitaires comme la leishmaniose et la trypanosomiase, ainsi que la rickettsiose et la peste. La plupart des articles utilisés dans notre étude combinaient la dynamique de transmission et la capacité vectorielle; seulement deux combinaient la dynamique de transmission et la co-infection. L'étude a identifié des lacunes de connaissances importantes au sujet du rôle des individus asymptomatiques, des effets de la co-infection et d'autres facteurs liés à l'hôte, ainsi que des impacts qu'ont les facteurs

climatiques, environnementaux et socio-économiques sur la transmission des maladies vectorielles dans les zones urbaines. Les limites comprenaient le fait de devoir affiner la stratégie de recherche (et de manquer de ce fait le modèle classique d'études), un manque d'études sur les co-infections - la plupart des études étant seulement descriptives et seules quelques unes offrant des recommandations en santé publique concrètes. Plus de recherches sur les risques de transmission dans les maisons et au travail sont nécessaires étant donné les populations de plus en plus dynamiques et mobiles. L'utilisation des réseaux sociaux présente un potentiel important pour la détection et le contrôle de plus larges modèles de transmission. Le manque d'études sur la co-infection a gêné la surveillance des infections transmises par le même vecteur.
Conclusions: Les stratégies principales identifiées pour les politiques et pratiques de santé publique sont un renforcement de la surveillance et du contrôle des maladies à transmission vectorielle, particulièrement pour les cas asymptomatiques et les populations mobiles, ainsi qu'une utilisation d'outils de détection rapide pour prédire une transmission croissante.

Translated from English version into French by Marie Piaget, proofread by Louis Gauvreau, through

TRANSLATORS
WITHOUT BORDERS

Обзорное исследование трансмиссивных заболеваний в городской среде: динамика распространения, показатели передачи и одновременное инфицирование

Маркус Эдер, Фанни Кортес, Ноэмия Тейксера де Сикиера Филха, Джиованни Винициус Араухо де Франса, Стефани Дегрот, Синтия Брага, Валери Ридд, Селина Мария Турчи Мартелли

Аннотация

Общие сведения: Динамика распространения, показатели передачи заболевания и одновременное инфицирование являются важными факторами заражения трансмиссивными заболеваниями городского и пригородного населения. Рассмотрение ключевых факторов заражения может дать представление о приоритетных областях исследований и помочь сформулировать рекомендации относительно потенциального вмешательства.
Основная часть: С помощью обзорного исследования необходимо выявить пробелы в информированности о динамике распространения, показателях передачи и одновременном инфицировании трансмиссивными заболеваниями в городских районах. Был проведен поиск среди рецензируемой и внеиздательской литературы, опубликованной между 2000 и 2016 годами. Нами были изучены рефераты и полные тексты работ с целью отобрать актуальные исследования. Посредством сетки экстракции была выделена общая информация, результаты, накопленный опыт и рекомендации, перспективы исследований и практические последствия. Мы сгруппировали исследования по трансмиссивным заболеваниям и странам/континентам и выявили соответствующие пробелы в знаниях. Из 773 выбранных для полнотекстового изучения статей в обзор вошли 50: из них 23 статьи основаны на исследованиях, проведенных в Северной и Южной Америках, 15 в Азии, 10 в Африке, и 1 статья была об исследованиях, проведенных в Европе и Австралии. Наибольшее количество данных о трансмиссивных заболеваниях в городской среде было собрано о лихорадке денге и малярии. Также были получены данные о таких антропонозных вирусах, как чикунгунья и вирус Западного Нила, паразитических заболеваниях лейшманиоз и трипаносомоз, бактериальный риккетсиоз и чума. В большинстве изученных нами статьях были одновременно рассмотрены динамика распространения и показатели передачи заболевания, и лишь в двух были рассмотрены

динамика распространения заболевания и одновременное инфицирование. В результате исследования были выявлено отсутствие данных о роли бессимптомных лиц, эффектах одновременного заражения и иных факторов организма-хозяина, влиянии климатических, экологических и социоэкономических факторов на распространение трансмиссивных заболеваний в городской среде. Среди причин ограниченности данного исследования можно выделить исключение классических моделей исследования в результате сужения стратегии поиска, недостаточную изученность одновременного заражения, описательный характер большинства исследований; лишь несколько статей предложили конкретные рекомендации по охране здоровья. Необходимо проведение исследований о рисках заражения дома и на рабочем месте, учитывая рост динамичности и мобильности населения. Использование социальных сетей предлагает большой потенциал для выявления и контроля за более широкими моделями передачи заболеваний. Отсутствие исследований об одновременном инфицировании препятствует мониторингу инфекций, передаваемых одним и тем же носителем.

Заключение: Ключевыми стратегиями здравоохранения являются усиление наблюдения за трансмиссивными заболеваниями, особенно в случаях бессимптомного протекания болезни и повышенной мобильности населения, а также использование инструментов раннего предупреждения для прогнозирования роста заболеваемости.

Translated from English version into Russian by Ekaterina Rugg, proofread by Natalia Potashnik, through

TRANSLATORS WITHOUT BORDERS

Revisión del alcance de las enfermedades transmitidas por vectores en áreas urbanas: dinámica de transmisión, capacidad vectorial y co-infección

Marcus Eder, Fanny Cortes, Noêmia Teixeira de Siqueira Filha, Giovanny Vinícius Araújo de França, Stéphanie Degroote, Cynthia Braga, Valéry Ridde, Celina Maria Turchi Martelli

Resumen

Antecedentes: La dinámica de transmisión, la capacidad vectorial y las co-infecciones poseen un impacto sustancial en las enfermedades transmitidas por vectores (ETV) que afectan a poblaciones urbanas y suburbanas. Una revisión de los factores clave puede revelar cuáles son las áreas de investigación prioritarias y ofrecer sugerencias para posibles intervenciones.

Cuerpo principal: Se realizó una revisión rigurosa a fin de identificar las brechas de conocimiento sobre la dinámica de la transmisión, la capacidad vectorial y las co-infecciones en lo que concierne a las ETV en áreas urbanas. Se revisaron los textos evaluados por pares y la "literatura gris", publicados entre 2000 y 2016. Examinamos cuidadosamente los resúmenes y textos completos para elegir los estudios. Por medio de una grilla de extracción recuperamos datos generales, resultados, lecciones aprendidas y recomendaciones, futuras líneas de investigación e implicaciones prácticas. Clasificamos los estudios según las ETS y el país /continente e identificamos las brechas de conocimiento relevantes. De los 773 artículos seleccionados para su examen completo, 50 se incluyeron en la revisión: 23 basados en investigaciones en las Américas, 15 en Asia, 10 en África y uno en Europa y Australia. La mayor evidencia existente sobre la epidemiología de las ETV en áreas urbanas se relaciona con el dengue y la malaria. Otros arbovirus examinados abarcaron la fiebre de chikungunya y el virus del Nilo Occidental, así como otras enfermedades parasitarias como la leishmaniasis y la tripanosomiasis, la

rickettsiosis bacteriana y la peste. La mayoría de los artículos rescatados en nuestra revisión combinaron la dinámica de transmisión y la capacidad vectorial; solo dos de ellos combinaron la dinámica de transmisión y la co-infección. Gracias a esta revisión se pudieron identificar brechas importantes del conocimiento sobre el rol de los individuos asintomáticos, los efectos de la co-infección y otros factores relativos al huésped, así como el impacto de los factores climáticos, ambientales y socioeconómicos en la transmisión de las ETV en áreas urbanas. Las limitaciones, debido a la estrategia de reducción en la búsqueda (omitiendo los estudios de modelos clásicos), implicaron la ausencia de estudios sobre co-infecciones. Además, la mayoría de los estudios resultaron puramente descriptivos y sólo unos pocos ofrecen recomendaciones concretas sobre salud pública. Debido a que las poblaciones son cada vez más dinámicas y móviles se necesitan mas investigaciones sobre el riesgo de transmisión en hogares y lugares de trabajo. El uso de las redes sociales ofrece un gran potencial para la detección y el control de patrones de transmisión más amplios. La falta de estudios sobre la co-infección obstaculiza el monitoreo de las infecciones transmitidas por el mismo vector.

Conclusiones: El aumento de la vigilancia y el control de las ETV, particularmente en casos asintomáticos y en poblaciones móviles, así como el uso de herramientas de alerta temprana para predecir el aumento de la transmisión, constituyeron estrategias clave para la identificación de políticas y prácticas de salud pública.

Translated from English version into Spanish by Guadalupe Barua, proofread byAngeles Possetti, through

TRANSLATORS
WITHOUT BORDERS
