# Supplementary material for: Scoping review on vector-borne diseases in urban areas: transmission dynamics, vectorial capacity and co-infection
Source: Infect Dis Poverty. 2018 Sep 3;7:90. doi: 10.1186/s40249-018-0475-7 (PMC6120094; doi:10.1186/s40249-018-0475-7)
Supplement: Supplementary file 2 — Literature search: Associated keywords, MESH and search terms. (DOCX 25 kb) [file 40249_2018_475_MOESM2_ESM.docx]

| **1.Transmission dynamics** | **2. Vectorial capacity** | **3. Co-infection** | **4. VBD** | **5. Urban areas** | **6.Impact on epidemiology** |
| --- | --- | --- | --- | --- | --- |
| transmission “basic reproduction number”  "basic reproductive number"  “basic reproductive ratio”  “basic reproductive rate”  immunity  “human  travel”  “human mobility” “human behaviour” | “vector capacity”  “vector competence”  “transgenic mosquit*  “genetically modified” “genetic engineering“ Wolbachia” | “Co- infection*”  “combined infection” “dual infection” “co circulation”  “Co-circulation”  “Multiple infections*” | "Chagas disease”  "encephalitis virus*"  "Lyme"  "phlebotomus fever*"  Rickettsia*  “Phlebovirus”  sandfly fever*  "sleeping sickness”  “Alphavirus*”  “Anaplasm*”  “Arbovirus*”  “arthropod*”  “Bartonellosis”  “Borreliosis”  “Borrelia”  “Chikungunya”  “Crimean-Congo”  “Dengue”  “Filariasis”  “Flavivirus*”  “Japanese Encephalitis”  “Leishmaniasis”  “malaria*”  “Onchocerciasis”  “plague”  “Rickettsiosis”  “rift valley fever”  “Tick-borne”  “trypanosome*"  “trypanosomiasis”  “typhus”  “west Nile fever”  “West Nile Virus”  “Yellow Fever”  “Zika”  "vector-borne"  haemorrhagic fever virus*  hemorrhagic fever virus*  "Relapsing fever"  "Yersinia pestis" | urban area*  urban population*  Urban*  municipal*  cities  city  neighbourhood*  neighbourhood* town*  borough* downtown* midtown*  megacit*  village*  cosmopolitan metropoli* megalopolis  slum*  suburb* | outbreak* epidemic*  morbidit*  prevalence  incidence  mortalit*  “case fatality” |

**PUBMED (MESH)**

(Transmission[Title/Abstract] OR "basic reproduction number"[Title/Abstract] OR "basic reproductive number"[Title/Abstract] OR "basic reproductive ratio"[Title/Abstract] OR "basic reproductive rate"[Title/Abstract] OR immunity[Title/Abstract] OR "human travel" [Title/Abstract] OR "human mobility" [Title/Abstract] OR "human behaviour" [Title/Abstract] OR "Disease Transmission, Infectious"[Mesh:NoExp] OR "Basic Reproduction Number"[Mesh] OR

"Infectious Disease Transmission, Vertical"[Mesh] OR "Immunity"[Mesh:noexp] OR "Population Dynamics"[Mesh]

OR

"vector capacity"[Title/Abstract] OR "vector competence"[Title/Abstract] OR "transgenic mosquito"[Title/Abstract] OR "transgenic mosquitoes"[Title/Abstract] OR "genetically modified"[Title/Abstract] OR "genetic engineering"[Title/Abstract] OR “Wolbachia"[Title/Abstract] OR "Genetic Engineering"[Mesh:NoExp] OR "Wolbachia"[Mesh]

OR

“Co-infection”[Title/Abstract] OR "Co- infections"[Title/Abstract] OR “Co infection”[Title/Abstract] OR “Co infections”[Title/Abstract] OR Coinfection*[Title/Abstract] OR "combined infection"[Title/Abstract] OR "combined infections"[Title/Abstract] OR "dual infection"[Title/Abstract] OR "dual infections"[Title/Abstract] OR "co-circulation"[Title/Abstract] OR "co circulation"[Title/Abstract] OR cocirculation[Title/Abstract] OR "Coinfection"[Mesh])

**AND**

"Chagas disease” [Title/Abstract] OR encephalitis virus* [Title/Abstract] OR "Lyme" [Title/Abstract] OR "phlebotomus fever"[Title/Abstract] OR Rickettsia* [Title/Abstract] OR Phlebovirus* [Title/Abstract] OR “sandfly fever” [Title/Abstract] OR "sleeping sickness” [Title/Abstract] OR Alphavirus* [Title/Abstract] OR Anaplasm* [Title/Abstract] OR Arbovirus* [Title/Abstract] OR arthropod [Title/Abstract] OR Bartonellosis [Title/Abstract] OR Borreliosis [Title/Abstract] OR Borrelia [Title/Abstract] OR Chikungunya [Title/Abstract] OR Crimean-Congo [Title/Abstract] OR Dengue [Title/Abstract] OR Filariasis [Title/Abstract] OR Flavivirus* [Title/Abstract] OR “Japanese Encephalitis” [Title/Abstract] OR Leishmaniasis [Title/Abstract] OR malaria [Title/Abstract] OR Onchocerciasis [Title/Abstract] OR plague [Title/Abstract] OR Rickettsiosis [Title/Abstract] OR “rift valley fever” [Title/Abstract] OR “Tick-borne” [Title/Abstract] OR trypanosome* [Title/Abstract] OR trypanosomiasis [Title/Abstract] OR typhus [Title/Abstract] OR “west Nile fever” [Title/Abstract] OR “West Nile Virus” [Title/Abstract] OR “Yellow Fever” [Title/Abstract] OR Zika [Title/Abstract] OR "vector-borne" [Title/Abstract] OR haemorrhagic fever virus* [Title/Abstract] OR hemorrhagic fever virus* [Title/Abstract] OR "Relapsing fever" [Title/Abstract] OR "Yersinia pestis"[Title/Abstract] OR "Chagas Disease"[Mesh:NoExp] OR "Trypanosomiasis, African"[Mesh] OR "Encephalitis Viruses"[Mesh] OR "Rickettsia Infections"[Mesh] OR "Alphavirus"[Mesh:NoExp] OR "Alphavirus Infections"[Mesh:NoExp] OR "Anaplasma"[Mesh] OR "Arbovirus Infections"[Mesh] OR "Arboviruses"[Mesh] OR "Chikungunya virus"[Mesh] OR "Chikungunya Fever"[Mesh] OR "Malaria"[Mesh] OR "Filariasis"[Mesh] OR "Leishmaniasis"[Mesh] OR "Trypanosoma"[Mesh] OR "Tick-Borne Diseases"[Mesh] OR Bartonellosis [Mesh] OR Borrelia Infections [Mesh] OR Hemorrhagic Fever, Crimean [Mesh] OR Kyasanur Forest Disease [Mesh] OR Plague [Mesh] OR Zika Virus [Mesh] OR "Yersinia pestis"[Mesh]

**AND**

“urban area” [Title/Abstract] OR “urban areas” [Title/Abstract] OR “urban population” [Title/Abstract] OR “urban populations” [Title/Abstract] OR Urban*  [Title/Abstract] OR municipal*  [Title/Abstract] OR cities  [Title/Abstract] OR city [Title/Abstract] OR neighborhood* [Title/Abstract] OR neighbourhood* [Title/Abstract] OR town* [Title/Abstract] OR borough* [Title/Abstract] OR downtown* midtown* [Title/Abstract] OR megacit* [Title/Abstract] OR village* [Title/Abstract] OR cosmopolitan [Title/Abstract] OR metropoli* [Title/Abstract] OR megalopolis [Title/Abstract] OR slum* [Title/Abstract] OR suburb* OR "Population Density"[Mesh] OR "Suburban Population"[Mesh] OR "Suburban Health"[Mesh] OR "Residence Characteristics"[Mesh] OR "Urbanization"[Mesh] OR "Urban Health"[Mesh] OR "Urban Population"[Mesh] OR "City Planning"[Mesh] OR "Cities"[Mesh]

**AND**

outbreak* [Title/Abstract] OR epidemic* [Title/Abstract] OR morbidit* [Title/Abstract] OR prevalence [Title/Abstract] OR incidence [Title/Abstract] OR mortalit* [Title/Abstract] OR “case fatality” OR "Disease Outbreaks"[Mesh] OR "Mortality"[Mesh] OR "Morbidity"[Mesh]

**EMBASE**

keywords

(transmission or basic reproduction number or basic reproductive number or basic reproductive ratio or basic reproductive rate or immunity or human travel or human mobility or human behaviour).ab,ti.

(vector capacity or vector competence or transgenic mosquit* or genetically modified or genetic engineering or Wolbachia).ab,ti.

(co-infection* or co infection* or coinfection* or co-circulation or co circulation or cocirculation combined infection or dual infection or multiple infection*).ab,ti.

(Chagas disease or encephalitis virus* or Lyme or phlebotomus fever* or Rickettsia* Phlebovirus or sandfly fever* or sleeping sickness or Alphavirus* or Anaplasm* or Arbovirus* or arthropod* or Bartonellosis or Borreliosis or Borrelia or Chikungunya or Crimean-Congo or Dengue or Filariasis or Flavivirus* or Japanese Encephalitis or Leishmaniasis or malaria* or Onchocerciasis or plague or Rickettsiosis or rift valley fever or Tick-borne or trypanosome* or trypanosomiasis or typhus or west Nile fever or West Nile Virus or Yellow Fever or Zika vector-borne or haemorrhagic fever virus* or hemorrhagic fever virus* or Relapsing fever or Yersinia pestis).ab,ti.

(urban area* or urban population* or urban* or municipal* or cities or city or neighborhood* or neighbourhood* or town* or borough* or downtown* or midtown* or megacit* or village* or cosmopolitan or metropoli* or megalopolis or slum* or suburb*).ab,ti.

(outbreak* or epidemic* or morbidit* or prevalence or incidence or mortalit* or case fatality).ab,ti.

**MESH**

bacterial transmission/ or disease transmission/ or vertical transmission/ or virus transmission/or exp basic reproduction number/ or immunity/ or virus immunity/ or bacterial immunity/ or herd immunity/

OR

mixed infection/

AND

Arbovirus/ or arthropod disease/ or lymphatic filariasis/ or [loiasis](http://ovidsp.tx.ovid.com/sp-3.21.1b/ovidweb.cgi?S=AIOEFPKBEMDDGDPDNCIKMHIBHMKFAA00&Controlled+Vocabulary=thes+loiasis&)/ or [mansonelliasis](http://ovidsp.tx.ovid.com/sp-3.21.1b/ovidweb.cgi?S=AIOEFPKBEMDDGDPDNCIKMHIBHMKFAA00&Controlled+Vocabulary=thes+mansonelliasis&)/ or [onchocerciasis](http://ovidsp.tx.ovid.com/sp-3.21.1b/ovidweb.cgi?S=AIOEFPKBEMDDGDPDNCIKMHIBHMKFAA00&Controlled+Vocabulary=thes+onchocerciasis&)/ or microfilariasis/ or exp malaria/ or exp flavivirus/ or flaviviridae infection/ or exp flavivirus infection/ or filariasis/ or anaplasmosis/ or exp trypanosomatid infection/ or exp leishmaniasis/ or exp trypanosomiasis/ or exp bartonellosis/ or exp rickettsiosis/ or [leishmaniasis](http://ovidsp.tx.ovid.com/sp-3.21.1b/ovidweb.cgi?&Controlled+Vocabulary=Mapping%7c0&Return=mapping&S=AIOEFPKBEMDDGDPDNCIKMHIBHMKFAA00)/ or exp rickettsiaceae infection/ or Rickettsia/ or typhus/ or exp nairovirus infection/ or exp phlebovirus infection/ or exp phlebovirus/ or plague/ or tick borne encephalitis/ or Crimean Congo hemorrhagic fever/ or tick borne disease/ or onchocerciasis/ or exp Borrelia infection/ or exp alphavirus/ or exp Alphavirus infection/ or exp encephalitis virus/ or Yersinia pestis/

AND

urban area/ or residential area/ or suburban area/ or urban population/ or housing/

AND

Epidemic/ or pandemic/ or epidemiology/ or mortality/ or morbidity/ or prevalence/ or incidence/

**GLOBAL HEALTH**

**MESH**

disease transmission/ or horizontal transmission/ or mechanical transmission/ or sexual transmission/ or transplacental transmission/ or vertical transmission/ or immunity/

OR

vector competence/ or vectorial capacity/ or exp genetic engineering/

OR

exp concurrent infections/ or exp mixed infections/

AND

outbreaks/ or epidemics/ or epidemiology/ or disease incidence/ or mortality/

AND

urban areas/ or urban environment/ or urban development/ or urban planning/ or urban population/ or urban sites/ or towns/ or neighbourhoods/ or residential areas/

AND

vector-borne diseases/ or exp filariasis/ or exp leishmaniasis/ or exp louse-borne typhus/ or exp mosquito-borne diseases/ or exp plague/ or exp sandfly fever/ or exp tickborne diseases/ or exp trypanosomiasis/ or exp dengue/ or exp japanese encephalitis/ or exp malaria/ or exp rift valley fever/ or exp yellow fever/ or exp bancroftian filariasis/ or exp filariasis perstans/ or exp lymphatic filariasis/ or exp onchocerciasis/ or exp cutaneous leishmaniasis/ or exp diffuse cutaneous leishmaniasis/ or exp mucocutaneous leishmaniasis/ or exp post kala azar dermal leishmaniasis/ or exp rickettsia prowazekii/ or exp typhus fevers/ or chagas' disease/ or exp rickettsial diseases/ or onchocerciasis/ or borrelia/ or exp relapsing fever/ or exp borrelia burgdorferi/ or exp tick-borne encephalitis virus/ or exp tickborne encephalitis/ or exp nairovirus/ or exp crimean-congo haemorrhagic fever virus/ or arboviruses/ or exp alphavirus/ or exp anaplasma/ or exp flavivirus/ or Yersinia pestis/

**COCHRANE (MESH)**

[transmission OR "basic reproduction number" OR "basic reproductive number" OR "basic reproductive ratio" OR "basic reproductive rate" OR immunity OR "human travel" or "human mobility" or "human behaviour" OR

Disease Transmission, Infectious OR Immunity OR Population Dynamics

OR

Vector capacity OR Vector competence OR Transgenic mosquito OR transgenic mosquitoes OR Genetically modified OR Wolbachia OR

Insecticide Resistance OR Animals, Genetically Modified OR Wolbachia

OR

Co-infection* OR Co infection* OR Coinfection* OR “combined infection” OR “dual infection” OR “co-circulation” OR “co circulation” OR “Cocirculation” “Multiple infection*” OR Coinfection ]

AND

"Chagas disease" OR encephalitis virus* OR "Lyme" OR "phlebotomus fever" OR Rickettsia* OR Phlebovirus* OR "sandfly fever" OR "sleeping sickness" OR Alphavirus* OR Anaplasm* OR Arbovirus* OR arthropod OR Bartonellosis OR Borreliosis OR Borrelia OR

Chikungunya OR Crimean-Congo OR Dengue OR Filariasis OR Flavivirus* OR "Japanese Encephalitis" OR Leishmaniasis OR malaria OR plague OR Rickettsiosis OR "rift valley fever" OR "Tick-borne" OR trypanosome* OR trypanosomiasis OR typhus OR "west Nile fever" OR "West Nile Virus" OR "Yellow Fever" OR Zika OR "vector-borne" OR haemorrhagic fever virus* OR hemorrhagic fever virus* OR "Relapsing fever" OR "Yersinia pestis" OR

[Chagas Disease](http://onlinelibrary.wiley.com/cochranelibrary/search/mesh?searchRow.searchOptions.conceptId=D014355&searchRow.searchCriteria.meshTerm=Chagas%20Disease&meshTreeSelect=true&searchRow.ordinal=0&hiddenFields.strategySortBy=last-modified-date;desc&hiddenFields.showStrategies=false) OR African Trypanosomiasis OR encephalitis viruses OR Lyme disease OR Q fever OR Rickettsia Infections OR Filariasis OR Borrelia infection OR Alphavirus infections OR Tick-Borne Diseases OR Arbovirus Infections OR Malaria OR plague

AND

"urban area*" or "urban population" or Urban* or municipal* or cities or city or neighborhood* or neighbourhood* town* or borough* or downtown* or midtown* or megacities or village* or cosmopolitan or metropoli* or megalopolis or slum* or suburb* OR

Urban Population OR Cities OR Residence Characteristics OR Poverty Areas OR Suburban OR Population OR Population Density

AND

outbreak* OR epidemic* OR morbidit* or prevalence or incidence or mortalit* or "case fatality" OR Disease Outbreaks OR Epidemics OR Morbidity OR Prevalence OR Incidence OR Mortality

**Grey literature**

Transmission* OR reproduct* OR immunit*) OR ((population* OR human*) NEAR/3 (travel* OR mobilit* OR dynamic* OR displacement*))) OR (Capacity OR Competence OR  "transgenic mosquitoes" OR "genetically modified" OR "genetic engineering"  OR wolbachia) OR  ("Co infection*" OR Coinfection*  OR “combined infection*” OR “dual infection*” OR "co circulation*" OR "co-circulation*")) AND (“Disease Vector*” OR “Vector-borne disease*” OR “Vectorborne disease*” OR “mosquito-borne disease*” OR “mosquito disease*” OR “zoonotic disease*” OR “zoonotic pathogen*” OR “vector-borne pathogen*” OR “Emerging disease*” OR “Arthropod-borne virus*” OR “arthropodborne disease*” OR zoonose* OR “communicable disease*” OR “neglected disease*” OR “tropical disease*” OR Arbovirus* OR Flavivirus* OR Dengue* OR Zika OR “West Nile Virus*” OR “west Nile fever*” OR “Japanese Encephalitis” OR “Yellow Fever*” OR Alphavirus* OR Chikungunya OR “encephalitis virus*” OR “paralytic virus*” OR malaria* OR filariasis OR leishmaniasis OR trypanosome* OR Bartonellosis OR typhus OR “rift valley fever*” OR “sandfly fever*” OR phlebotomus fever* OR “Chagas disease*”  OR trypanosomiasis OR “Sleeping sickness” OR Plague* OR Rickettsiosis OR Onchocerciasis OR “Relapsing fever*” OR borreliosis OR “Rickettsial disease*” OR “Tick-borne encephalitis” OR “Crimean-Congo haemorrhagic fever*” OR “Lyme disease*” OR “yersinia pestis” OR haemorrhagic fever virus* OR hemorrhagic fever virus* OR “anaplasm”) AND (outbreak* OR epidemi* OR morbidit* OR prevalence OR incidence OR mortalit* OR "case fatality") AND (urban* OR Municipal* OR cities OR city OR neighborhood* OR neighbourhood* OR town* OR borough* OR downtown* OR midtown* OR megacit* OR village* OR cosmopolitan OR metropoli* OR megalopolis OR slum* OR district* OR suburb*
