# Supplementary material for: Scoping review on vector-borne diseases in urban areas: transmission dynamics, vectorial capacity and co-infection
Source: Infect Dis Poverty. 2018 Sep 3;7:90. doi: 10.1186/s40249-018-0475-7 (PMC6120094; doi:10.1186/s40249-018-0475-7)
Supplement: Supplementary file 3 — Final reference selection by country. (DOCX 15 kb) [file 40249_2018_475_MOESM3_ESM.docx]

| **Country** | Malaria | Leishmaniasis | Chagas disease | Dengue | West Nile virus | Chikungunya | Yellow fever | Ross River virus | Plague | Rickettsiae |
| --- | --- | --- | --- | --- | --- | --- | --- | --- | --- | --- |
| Argentina |  | 1 |  | 1 |  |  |  |  |  |  |
| Bolivia |  |  | 1 |  |  |  |  |  |  |  |
| Brazil |  | 2 |  | 5 |  |  | 1 |  |  | 1 |
| Colombia |  |  |  | 1 |  |  |  |  |  |  |
| Cuba |  |  |  | 1 |  |  |  |  |  |  |
| French Guiana | 1 |  |  | 1 |  |  |  |  |  |  |
| Mexico |  |  | 1 | 1 |  |  |  |  |  |  |
| Puerto Rico |  |  |  | 1 |  |  |  |  |  |  |
| USA |  |  |  | 1 | 2 |  |  |  |  |  |
| Venezuela | 1 |  |  |  |  |  |  |  |  |  |
| **Americas** | 2 | 3 | 2 | 12 | 2 | 0 | 1 | 0 | 0 | 1 |
| Italy |  |  |  |  |  | 1 |  |  |  |  |
| **Europe** | 0 | 0 | 0 | 0 | 0 | 1 | 0 | 0 | 0 | 0 |
| Burkina Faso | 1 |  |  |  |  |  |  |  |  |  |
| Ethiopia | 2 |  |  |  |  |  |  |  |  |  |
| Mali | 1 |  |  |  |  |  |  |  |  |  |
| Nigeria | 1 |  |  |  |  |  |  |  |  |  |
| Rwanda | 1 |  |  |  |  |  |  |  |  |  |
| São Tomé | 1 |  |  |  |  |  |  |  |  |  |
| Sudan | 1 |  |  | 1 |  | 1 |  |  |  |  |
| **Africa** | 8 | 0 | 0 | 1 | 0 | 1 | 0 | 0 | 0 | 0 |
| Bangladesh |  |  |  | 1 |  |  |  |  |  |  |
| China | 2 |  |  | 3 |  |  |  |  |  |  |
| India | 2 |  |  |  |  |  |  |  |  |  |
| Nepal |  | 1 |  |  |  |  |  |  |  |  |
| Singapore | 1 |  |  |  |  | 1 |  |  |  |  |
| Sri Lanka |  |  |  |  |  |  |  |  |  |  |
| Taiwan |  |  |  | 2 |  |  |  |  |  |  |
| Vietnam |  |  |  | 1 |  |  |  |  | 1 |  |
| **Asia** | 5 | 1 | 0 | 7 | 0 | 1 | 0 | 0 | 1 | 0 |
| Australia |  |  |  |  |  |  |  | 1 |  |  |
| **Australia** | 0 | 0 | 0 | 0 | 0 | 0 | 0 | 1 | 0 | 0 |
| **Total (n = 51)** | 15 | 4 | 2 | 20 | 2 | 3 | 1 | 1 | 1 | 1 |
